# Identification of new pharmacophore against SARS-CoV-2 spike protein by multi-fold computational and biochemical techniques

**DOI:** 10.1038/s41598-024-53911-6

**Published:** 2024-02-13

**Authors:** Atta Ullah, Saeed Ullah, Sobia Ahsan Halim, Muhammad Waqas, Basharat Ali, Farid S. Ataya, Nasser M. El-Sabbagh, Gaber El-Saber Batiha, Satya Kumar Avula, Rene Csuk, Ajmal Khan, Ahmed Al-Harrasi

**Affiliations:** 1 Natural and Medical Sciences Research Center, University of Nizwa, Birkat-Ul-Mouz, P.O Box 33, Postal Code 616 Nizwa, Sultanate of Oman; 2 Sulaiman Bin Abdullah Aba Al-Khail-Centre for Interdisciplinary Research in Basic Sciences (SA-CIRBS), International Islamic University, Islamabad, Pakistan; 3 Department of Biochemistry, College of Science, King Saud University, PO Box 2455, 11451 Riyadh, Saudi Arabia; 4 Department of Veterinary Pharmacology, Faculty of Veterinary Medicine, Alexandria University, Edfina, Egypt; 5 Department of Pharmacology and Therapeutics, Faculty of Veterinary Medicine, Damanhour University, Damanhour, 22511 AlBeheira Egypt; 6 Organic Chemistry, Martin-Luther-University Halle-Wittenberg, Kurt-Mothes-Str. 2, 06120 Halle (Saale), Germany

**Keywords:** SARS CoV-2, Spike protein, Molecular docking, In-vitro assay, Boswellic acid, Chemical biology, Computational biology and bioinformatics

## Abstract

COVID-19 appeared as a highly contagious disease after its outbreak in December 2019 by the virus, named SARS-CoV-2. The threat, which originated in Wuhan, China, swiftly became an international emergency. Among different genomic products, spike protein of virus plays a crucial role in the initiation of the infection by binding to the human lung cells, therefore, SARS-CoV-2’s spike protein is a promising therapeutic target. Using a combination of a structure-based virtual screening and biochemical assay, this study seeks possible therapeutic candidates that specifically target the viral spike protein. A database of ~ 850 naturally derived compounds was screened against SARS-CoV-2 spike protein to find natural inhibitors. Using virtual screening and inhibitory experiments, we identified *acetyl 11-keto-boswellic acid* (AKBA) as a promising molecule for spike protein, which encouraged us to scan the rest of AKBA derivatives in our in-house database via 2D-similarity searching. Later 19 compounds with > 85% similarity with AKBA were selected and docked with receptor binding domain (RBD) of spike protein. Those hits declared significant interactions at the RBD interface, best possess and excellent drug-likeness and pharmacokinetics properties with high gastrointestinal absorption (GIA) without toxicity and allergenicity. Our *in-silico* observations were eventually validated by in vitro bioassay, interestingly, 10 compounds **(A3, A4, C3, C6A, C6B, C6C, C6E, C6H, C6I,** and **C6J)** displayed significant inhibitory ability with good percent inhibition (range: > 72–90). The compounds **C3** (90.00%),** C6E** (91.00%), **C6C** (87.20%), and **C6D** (86.23%) demonstrated excellent anti-SARS CoV-2 spike protein activities. The docking interaction of high percent inhibition of inhibitor compounds **C3** and **C6E** was confirmed by MD Simulation. In the molecular dynamics simulation, we observed the stable dynamics of spike protein inhibitor complexes and the influence of inhibitor binding on the protein’s conformational arrangements. The binding free energy ΔG_TOTAL_ of **C3** (−38.0 ± 0.08 kcal/mol) and **C6E** (−41.98 ± 0.08 kcal/mol) respectively indicate a strong binding affinity to Spike protein active pocket. These findings demonstrate that these molecules particularly inhibit the function of spike protein and, therefore have the potential to be evaluated as drug candidates against SARS-CoV-2.

## Introduction

A novel coronavirus infection epidemic (COVID-19) was identified in December 2019 in Wuhan city, Hubei province, when the first case manifested with pneumonia-like symptoms in seafood and live animal markets in China ^1^. The symptoms of this disease in patients included fever, difficulty in breathing, malaise, dry cough, and dyspnea^2^. Talking, coughing, and sneezing contribute to the spread of COVID-19 because infected people expel infectious virus particles into the air^3,4^. *Betacoronavirus*, the genus to which SARS-CoV-2 belongs, is a member of the family *Coronaviridae*. Also included in the genus *Betacoronavirus* are other human coronaviruses like SARS-CoV (which caused the SARS outbreak in 2003) and MERS-CoV. (Responsible for the Middle East respiratory syndrome outbreak in 2012)^5^.

Coronaviruses are non-segmented, encapsulated viruses that have 26–32 kb of single-stranded RNA (ssRNA). The transmembrane spike (S) glycoprotein, projecting from the viral surface, mediates coronavirus entrance into host cells. Spike (S) comprises two functional subunits, S1 and S2, that collaborate to connect to the angiotensin-converting enzyme 2 (ACE2) receptor on the host cell and fuse the viral and cellular membranes (S2 subunit)^6^. After attaching to ACE2 in the “open” conformation, one or more receptor binding domains (RBDs) of S-protein acquire “up” conformation, thus, the virus particle can fuse with the human host cell membrane by a process known as cleavage into S1 and S2 subunits^7^. TM protease serine 2 (TMPRSS2), a type 2 TM serine protease localized on the host cell membrane, activates the Spike protein upon binding to the receptor, hence facilitating viral entrance into the cell^8^. As the virus enters the human lung cell, they release its RNA and use the host machinery to make its copies^9^. Attachment of the SARS-CoV-2 virus to host cell receptors requires the RBD S1 subunit of spike protein, therefore by specifically inhibiting this subunit, virus's access to the host cell can be restricted, and consequently viral genome's replication within the host cell can be impeded^10^. SARS-CoV-2 antibodies that have been discovered primarily target the trimeric S glycoproteins, and most of them can recognize epitopes in the RBD that bind the ACE2 receptor^11^. Additional drugs, that target the Spike protein RBD-ACE2 interface and obstruct this protein–protein interaction, could be COVID-19 treatment options^12,13^. To combat this highly contagious infectious agent, researchers may search for new compounds that can precisely block the RBD and *h*ACE2 interaction using bioinformatics tools, in silico methods, and inhibition assay^14,15^. Up to date there is, no specific medications that have been approved to treat the distinct coronavirus infection. However, clinical trials of synthetic drug compounds have been started to evaluate coronavirus treatments^16^. Mostly synthetics drugs in clinical trials are inhibiting the viral protein ^17^. Overcoming the issue of SARS-CoV-2 genome change and viral resistance to medicines and vaccines over time remains a challenge^18–21^.

**Figure 1:**
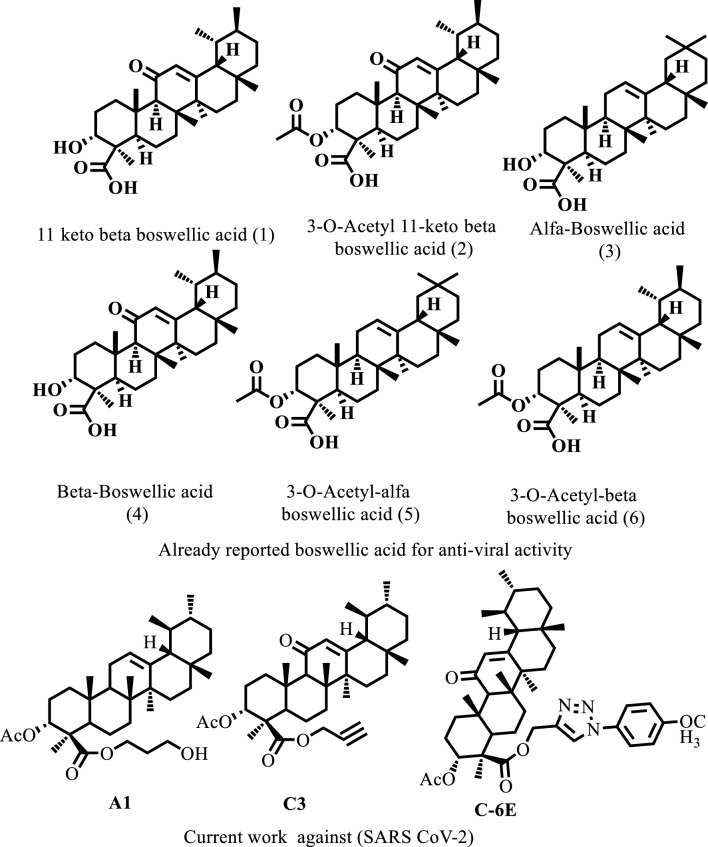
Some already reported compounds for anti-viral activities.

To get beyond this obstacle small, naturally occurring substances with inhibitory properties could prevent the SARS CoV-2 spike protein from interacting with the hACE2 receptor^22,23^. When compared to synthesized medications, naturally occurring or derived substances typically have less side effects and are more effective in blocking enzymes and proteins^24^. Natural substances have been refined by the elements to engage specifically with biological targets while having a low immunological response and good absorption^25^. So, this study aims to discover novel potential inhibitors against SARS CoV-2 spike protein from naturally derived compound (AKBA) (Fig. 1) derivatives by applying 2D similarities searching via computational tools and testing by inhibition assay, which is a crucial step of drug discovery. The AKBA is a naturally derived compound from *Boswellia serrata* and have uses against antiviral and chronic inflammatory diseases of the lung ^26,27^. The inhibition activity of AKBA is reported for many diseases (Brain, Lungs, and Viral infection)^28–31^. So based on potent inhibition activity of AKBA in this study we aim to discover potential inhibitors from its derivatives against spike protein receptor binding domain.

## Material and methods

### Structure retrieval of SARS CoV-2 spike protein

The reported crystal structure of spike protein receptor binding domain (RBD) bound with human ACE2 receptor (6M0J, resolution = 2.45 Å) was obtained from Protein Data Bank (RCSB) (https://www.rcsb.org/)^32^. Molecular Operating Environment (MOE) version 2022.02 built loop modeler algorithm with Amber14: EHT (Amber ff14SB combined with the EHT) forcefield was applied to model the missing residues in the 3D structure of RBD^33^. With the help of MOE’s Loop builder feature, we added the missing loops to the spike protein chain, and important terminals like start and end (C-N) were labeled^34^. The MOE’s Quick Prep module modified the protein file by adding missing parameters (hydrogen bonds, angle parameters, partial charges, and Vander Wal forces) to the receptor binding domain^35^ using Amber: EHT14 forcefield^36^.

### Screening of inhibitors candidates by docking

**Figure 2:**
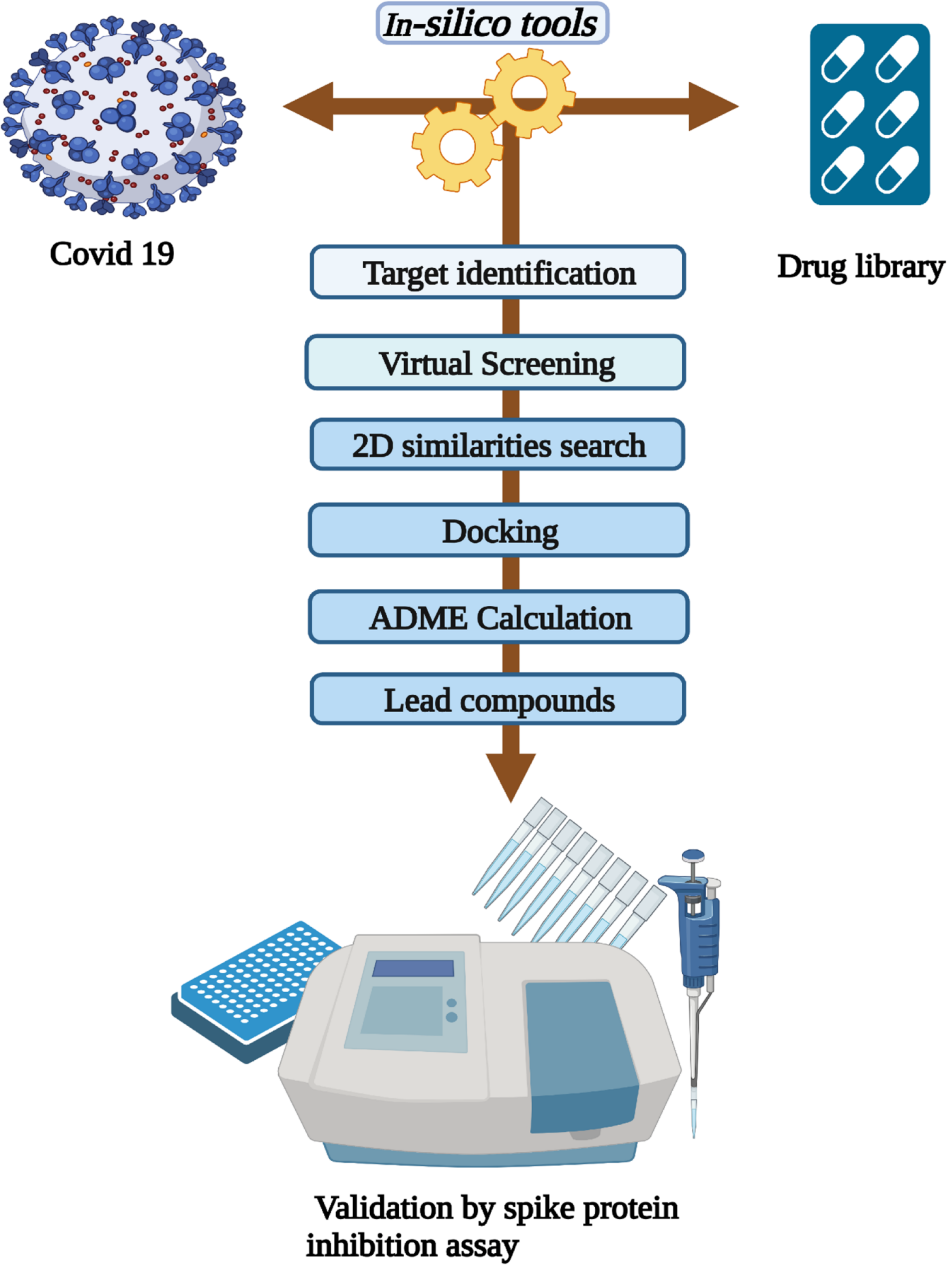
Schematic workflow of design study.

For searching inhibitors from natural sources, an in-house collection of ~ 850 compounds was docked at the receptor binding domain using MOE Dock tool^37^. Preceding docking, partial charges and hydrogen were added in each compound in the database via MMFF94x forcefield in MOE, and their structures were minimized with RMS gradient of 0.5 kcal/mol/Å^38^. The docking site for compounds was selected from the protein–protein interface where the human ACE2 receptor binds with the Spike protein RBD domain. We applied the MOE's Triangle Matcher approach^39^ and the London dG Scoring system^40^ with optimal 100 postures, and the GBVI-WSA dg scoring technique was used to determine the final 30 postures for each chemical. Afterward, the docked library was ranked based on the top docking score (i.e., highly negative) and binding position. The binding interactions of compounds at RBD were quantitively calculated by Protein–Ligand Interaction Fingerprints (PLIF) of MOE^41^. The schematic workflow of this study is presented in Fig. 2.

### 2D-similarity searching

Comparison of fingerprints is the most common approach to discover the structural similarities between compounds. Because of good interactions and top docking score, 3-acetyl 11-keto-β-boswellic acid (AKBA) was taken to search its structurally similar compounds using 2D similarity searching approach. To calculate the 2D-similarity searches for AKBA, the “Tanimoto coefficient” ^42^ and MACCS (Molecular Access System) structural keys (166 bits of MOE)^43^ was used with > 85% cutoff. First, we calculate the MACCS fingerprint for all compounds in the in-house database and search the fingerprints for similar compounds, then those with > 85% similarity with AKBA were selected.

### Estimation of drug-like characteristics and pharmacokinetics of compounds

The drug-like behavior of compounds, as well as their physiochemical characteristics and pharmacokinetics, were calculated by a server SwissADME^44^ to study the compounds’ modes of absorption, toxic profiles, metabolic pathways, and distribution^45^. By using ProTox-II server^46^, various toxicity parameters of compounds, such as rat oral median lethal dose (LD50), hepatotoxicity, carcinogenicity, mutagenicity, immunotoxicity, and cytotoxicity were predicted. The canonical SMILES (Simplified Molecular Input Line Entry System) of each compound was supplied to the servers to compute the relevant properties. The allergenicity of selected compounds was predicted using the server CHAIPred^47^ because some of the small inhibitors after binding to a specific protein can elicit immune response^48^.

### Spike protein inhibition bioassay

The selected natural compounds obtained through virtual screening and by 2D similarity searches were tested by inhibitory assay of the spike protein. By using the Spike protein kit assay (BPS Bioscience/Tebu-bio, Offenbach, Germany), the inhibitory efficacy of drugs was measured following the described procedure^49^. A colorimetric assay was used to evaluate the strength of the interaction between RBD and ACE2^50^. The assay was started by adding 50 µL of ACE2/well into a 96-well plate followed by an hour of incubation at room temperature (25 °C) with gentle shaking. After one hour, the 96-well plate was washed 3 times with 1× immune-buffer 1, 100 µL of buffer/well, followed by adding 100 µL blocking buffer-2/well and incubated at room temperature (25 °C) with shaking for 10 min, and later washed with the same washing buffer 1. All the compounds were dissolved in 10% DMSO and tested at a concentration of 0.5 mM. Additionally, as a positive control, 30 µL of blocking buffer 2 was utilized in each well. Moreover, a 96-well plate with 50 µL of blocking buffer 2 in each well was used as a blank and incubated for an hour at ambient temperature. The test (compounds) wells and the positive control wells received 20 µL of spike S1 (0.0625 n/µL) each well after the third time incubation. The 96-well plate was incubated once more for an hour at 25 °C while being shaken, after which 100 µL of antibody 1 that had been HRP-labeled (dissolved in blocking buffer 2) was added to each well and incubated for an hour at room temperature (25 °C), after which the plate was washed with washing buffer 1. Ultimately, 100 µL of colorimetric HRP substrate per well were employed and incubated at room temperature until the positive control turned blue^51^. The reading was taken at 450 nm after the blue color developed and 100 µL of 1N HCl had been added to each well. The following calculation was used to calculate the percent inhibition using the SoftMax Pro and Excel software programs.

1 $$\begin{array}{*{20}c} {\% Inhibition = 100 - \left( {\frac{{O.D_{test \;compound} }}{{O.D_{control} }}} \right) \times 100} \\ \end{array}$$

### Molecular dynamic simulations

The compounds which show high inhibition rate were further subjected to MD simulation to confirm their binding stability with protein. For this MD simulation, we chose a reference-inhibited system spike protein receptor binding domain bound with hACE2 receptor PDB ID (6MOJ). The AMBER22 software package was used to run molecular dynamics simulations^52^. Topology and coordinate files for each system were generated in AMBER22’s LEap module using the ff19SB residue-specific force field^53^. To simplify the simulation of protein–ligand interactions, the ff19SB force field parameterized the residues of the protein. Monovalent OPC ions (Na+ and Cl−) were added to each system and the NI++ elements were treated with the divalent OPC ion library to maintain electrostatic neutrality in the systems^54^. To get reliable simulation results, it is necessary to neutralize the system’s charge by adding counterions. When it comes to creating an optimal environment for protein–ligand interactions, water molecules play a vital role. Because of their importance to the simulations' hydration and stability, an octahedral OPC-water model was used to solvate the systems. A buffer area comprised of water molecules extending out to 12 Å from the protein surface was created. Prior to the MD production run, the minimum energy state was achieved by a two-step energy reduction method. By reorganizing the atoms in the system, we can reduce the amount of stress and steric conflicts that exist^36^. The steepest descent algorithm, a gradient-based optimization method, was initially applied for 50,000 steps^55^. It iteratively adjusts the positions of the atoms in the system by following the negative gradient of the potential energy until a local minimum is reached. After the initial steepest descent minimization, another 25,000 iterations with the conjugate gradient approach were performed^52^. Another optimization method that quickly reaches a local minimum by merging data from earlier iterations is the conjugate gradient method. During the process of minimizing the energy, the water molecules were free to move while the protein residues were held in place. This calms the protein–ligand complex by allowing water molecules to equalize around the protein. Before the dynamic production runs, the systems must be gradually equilibrated at the desired temperature and pressure. Using a Langevin thermostat, the systems were gradually heated from a lower temperature to the target simulation temperature (300 K) at 400 ps^52^. The Langevin thermostat maintains the system heated by subjecting them to random forces that are supposed to represent their interaction with a heat bath. During heating, the kinetic energy was adjusted for dynamic propagation using the Langevin thermostat with a collision frequency value of 2.0 ps for the harmonic oscillators. This makes for an easily achieved target temperature in the system. Any potential steric conflicts were weeded out by the SHAKE algorithm before the dynamic production run^55^. SHAKE is a constraint algorithm that keeps hydrogen-atom covalent bonds stable, letting the simulation run for a longer period. After heating, density adjustment was performed in a 400 ps run with the same protocol. This permits water molecules to settle around the protein–ligand complex and completes the equilibration of the system at the desired temperature. Subsequently, each system was equilibrated for 5000 ps at 300 K under the NPT ensemble, which means the number of particles (N), Pressure (P), and Temperature (T) were kept constant. During this equilibration, no restrictions were placed on the arrangement of the atoms, so the system was free to investigate all possible phases. The production MD run, the meat of the simulations in which data for analysis and insights into protein–ligand interactions are obtained, could begin once the systems had equilibrated. Each system underwent a production run of 200 ns^56^. This duration of simulation is optimized for capturing the protein–ligand complex’s dynamics and producing statistically significant results^57^. An 8 Å cutoff distance was utilized in order to compute non-bonded interactions, such as van der Waals and electrostatic interactions, efficiently. Beyond this point, interactions are ignored, which allows the simulation to run faster without compromising accuracy. At 10 ps, all system’s trajectory was observed throughout the production phase. These trajectories capture the atomic positions and velocities of the protein and inhibitors ligand pair, which can be examined to determine the changes in the system.

### MD production evaluation

CPPTRAJ, a tool in AMBER 22, was used to examine the simulated trajectories at 1 ps intervals^58^. Root Mean Square Fluctuation (RMSF) and Root Mean Square Deviation (RMSD) were computed for all systems containing Cα atoms using Eqs. (2) and (3).

2 $$\begin{array}{*{20}c} {RMSD = \sqrt {\frac{{\mathop \sum \nolimits_{i = 0}^{N} \left[ { m_{i} *\left( {X_{i} - Y_{i} } \right)^{2} } \right]}}{M}} } \\ \end{array}$$

In RMSD calculation, N = the number of atoms, mi = mass of atoms, Xi = the target atom i vector coordinate, Yi = the reference atom i vector coordinate, and M = the total mass.

3 $$\begin{array}{*{20}c} {RMSF\left( i \right) = \sqrt { \left\langle {\left( {x_{i} - \left\langle {x_{i} } \right\rangle } \right.)^{2} } \right\rangle } } \\ \end{array}$$

By averaging the positions of the atoms in the input frames denoted by x, we were able to get the RMSF of the atom i of interest. Using the (Rg) Radius of gyration, we were able to calculate the locations and velocities of the atoms at each time step^59^.

### Principal component analysis

Using AMBER 22’s CPPTRAJ module, principal component analysis (PCA) was used to determine the protein’s slow and efficient motions. First, Eq. (4) gives us the predicted covariance matrix C, and its component Cij.

4 $$\begin{array}{*{20}c} { C_{ij} = \left\langle {\left( {x_{i} } \right. - \left. {\left\langle {x_{j} } \right\rangle } \right)} \right\rangle \left\langle {\left( {x_{i} } \right. - \left. {\left\langle {x_{j} } \right\rangle } \right)} \right\rangle } \\ \end{array}$$

C atoms’ ith and jth cartesian coordinates are designated by xi and xj, respectively; the average ith and jth atom’s coordinates during the duration of the ensemble are written as x_i_ and x_j_, respectively.

The location-based covariance matrix of each system was computed using the 3-D spatial parameters of each system’s production trajectory on a time scale with ten motion patterns. Principal Components were obtained by firstly computing the covariance matrix C. The eigenvalue and eigenvector of the matrix were then calculated after the matrix was diagonalized. The resultant principal components (PCs) characterize the motion descriptively, whereas the eigenvalues quantify that description The proportional impact of each eigenvector was included. To examine their changes, the first two PCs were displayed on a graph.

### Characterization of hydrogen bonds

Here, we used the hbond function in CPPTRAJ to look at the interactions between the targeted Spike protein and the molecules of concern. By examining all the system’s 200 ns excursion, the typical length, their distance, and angle of the hydrogen bonds formed with the object being targeted and ligand were identified. The bond angle between the protein–ligand’s electron acceptor and donor atoms was fixed at 120 degrees and the cutoff distance was 3.5 Å^60^.

### Binding free energy calculations

The Generalized Born accessibility algorithms (MMGB-SA) implemented in AMBER22 were used to calculate the binding free energy of the selected inhibitors concerning the Spike protein RBD^61^. Equation (5) was used to get the free energy of the Spike protein complex and its free APO form.

5 $$\begin{array}{*{20}c} {\Delta G_{bind} = G_{R + L} - \left( {G_{R} + G_{L} } \right)} \\ \end{array}$$

The combined energy of the protein and the inhibitor, denoted by “GR + L,” and the free energy of binding, designated by “Gbind,” is represented by Eq. (6).

6 $$\begin{array}{*{20}c} {\Delta G = E_{bond} + E_{VDW} + E_{elec} + G_{GB} + G_{SA} - TS_{S} } \\ \end{array}$$

Equation (4) shows the components of the two systems’ total energy “ΔG”. The “E_bond_” term represents the energy associated with the bonds and dihedral angles, while “E_vdw_” represents van der Waals energy. “E_elec_” signifies the electrostatic energy, and “G_GB_” and “G_SA_” represent the contributions of the polar and non-polar solvents (usually water). Finally, “T” represents the absolute temperature of both systems, and S_S_ is used to calculate the protein's entropy.

### Data analysis

MOE 2022.02 and Blender were used to create graphs and visuals. Origin-Pro was used to extract the ensemble of lowest energy structures for each system, and those structures were then utilized to generate all graphs.

## Results

### Structural details of receptor binding domain of spike protein

**Figure 3:**
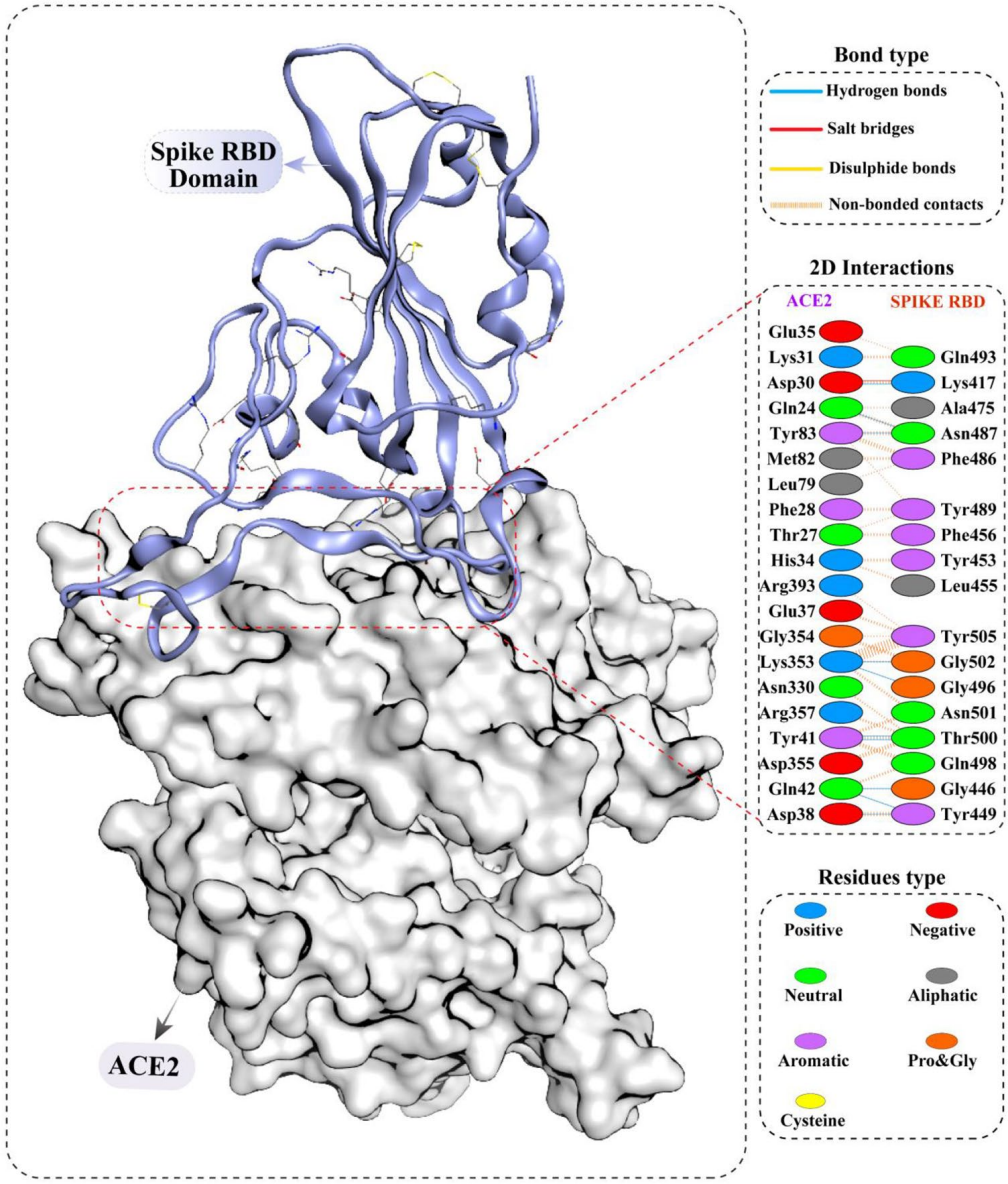
The 2D interactions diagram shows the crystal structure of the SARS-CoV-2 spike protein attached to human ACE2 receptor (PDB ID:6M0J).

The receptor binding domain (RBD) of the SARS CoV-2 spike protein (PDB ID: 6M0J) was taken from the protein databank RCSB, and the in-house compound’s database was docked at the protein–protein interface where RBD interacts with the *h*ACE2 receptor. The residues of the *h*ACE2 receptor (GLN24, ASP30, ASP38, TYR41, TYR41, GLN42, GLN42, TYR83, LYS353, LYS353, and ASP30) interact with RBD of spike protein (residues: ASN487, LYS417, TYR449, THR500, THR500, GLY446, TYR449, ASN487, GLY502, GLY496 and LYS417) to stabilize protein–protein interaction through hydrogen bonds and salt bridges. The detailed interaction of spike protein and *h*ACE2 receptor is given in (Fig. 3).

### Selection of inhibitors by virtual screening and 2D-similarity searching

The virtual screening of ~ 850 compounds was performed at the active pocket of SARS CoV-2 spike RBD. To select the natural products, the docked library was ordered by its docking score, and acetyl 11-keto-β-boswellic acid (AKBA) was identified among the top listed compounds with the highest docking score and good docked position. Therefore, AKBA was tested against spike protein in the in-vitro inhibition assay, where AKBA demonstrated significant percentage inhibition against RBD (80.56%). This in-vitro result compelled us to search other AKBA-like compounds in the in-house database, therefore 2D-similarity searching was conducted by MOE using the MACCS structural keys and a Tanimoto coefficient ^51^. There are 166 different structural substructures or patterns in MACCS made up of atoms other than hydrogen. The default value of 85% overlap was chosen for this search. The inhibitor AKBA with good inhibition against spike protein was selected as a query. By 2D-similarity search, we obtained 19 derivatives of acetyl 11-keto-β-boswellic acid (AKBA) (Table 1), their binding at RBD was examined, and their physicochemical properties and drug-likeness was estimated.

**Table 1:** Chemical structures of the selected hits for in-vitro testing.

Code	Structure	Names
AKBA		acetyl 11-keto-boswellic acid
A1		3-hydroxypropyl (3R,4R,6aR,6bS,8aR, 11R,12S,12aR,14bR)-3-acetoxy-4,6a, 6b,8a,11,12,14b-heptamethyl-1,2,3,4,4a, 5,6,6a,6b,7,8,8a,9,10,11,12,12a,14,14a,14b -icosahydropicene-4-carboxylate
A2		3-(tosyloxy)propyl (3R,4R,6aR,6bS,8aR,11R, 12S,12aR,14bR)-3-acetoxy-4,6a,6b,8a,11,12, 14b-heptamethyl-1,2,3,4,4a,5,6,6a,6b,7,8,8a, 9,10,11,12,12a,14,14a,14b-icosahydropicene -4-carboxylate
A3		3-azidopropyl (3R,4R,6aR,6bS,8aR,11R,12S, 12aR,14bR)-3-acetoxy-4,6a,6b,8a,11,12,14b- heptamethyl-1,2,3,4,4a,5,6,6a,6b,7,8,8a,9, 10,11,12,12a,14,14a,14b-icosahydropicene -4-carboxylate
A4		3-(4-phenyl-1H-1,2,3-triazol-1-yl) propyl (3R,4R,6aR,6bS,8aR,11R,12S,12aR,14bR) -3-acetoxy-4,6a,6b,8a,11,12,14b-heptamethyl -1,2,3,4,4a,5,6,6a,6b,7,8,8a,9,10,11,12,12a,14, 14a,14b-icosahydropicene-4-carboxylate
A5		3-(4-(4-(trifluoromethyl) phenyl)-1H-1,2,3-triazol-1-yl) propyl (3R,4R,6aR,6bS,8aR,11R,12S,12aR,14bR)-3 -acetoxy-4,6a,6b,8a,11,12,14b-heptamethyl-1,2,3,4,4a, 5,6,6a,6b,7,8,8a,9,10,11,12,12a,14,14a,14b -icosahydropicene-4-carboxylate
A6		3-(4-(4-fluorophenyl)-1H-1,2,3-triazol-1-yl) propyl(3R,4R,6aR,6bS,8aR,11R,12S,12aR,14bR) -3-acetoxy4,6a,6b,8a,11,12,14bheptamethyl-1,2,3,4,4a ,5,6,6a,6b,7,8,8a,9,10,11,12,12a,14,14a,14b- icosahydropicene-4-carboxylate
A7		methyl 1-(3-(((3R,4R,6aR,6bS,8aR,11R,12S,12aR, 14bR)-3-acetoxy-4,6a,6b,8a,11,12,14b-heptamethyl- 1,2,3,4,4a,5,6,6a,6b,7,8,8a,9,10,11,12,12a,14, 14a,14b-icosahydropicene-4-carbonyl) oxy)propyl) -1H-1,2,3-triazole-4-carboxylate
C3		prop-2-yn-1-yl (3R,4R,6aR,6bS,8aR,11R,12S,12aR,14bS) -3-acetoxy-4,6a,6b,8a,11,12,14b-heptamethyl- 14-oxo-1,2,3,4,4a,5,6,6a,6b,7,8,8a,9,10,11,12,12a, 14,14a,14b-icosahydropicene-4-carboxylate
C6A		(1-phenyl-1H-1,2,3-triazol-4-yl) methyl (3R,4R,6aR,6bS,8aR,11R,12S,12aR,14bS)-3 -acetoxy-4,6a,6b,8a,11,12,14b-heptamethyl-14-oxo- 1,2,3,4,4a,5,6,6a,6b,7,8,8a,9,10,11,12,12a,14,14a, 14b-icosahydropicene-4-carboxylate
C6B		(1-(o-tolyl)-1H-1,2,3-triazol4yl) methyl (3R,4R,6aR,6bS,8aR,11R,12S,12aR,14bS) 3acetoxy4,6a,6b,8a,11,12,14bheptamethyl1 4oxo1,2,3,4,4a,5,6,6a,6b,7,8,8a,9,10,11, 12,12a,14,14a,14b-icosahydropicene -4-carboxylate
C6C		(1-(2-methoxyphenyl)-1H-1,2,3-triazol-4-yl) methyl (3R,4R,6aR,6bS,8aR,11R,12S,12aR,14bS)-3-acetoxy -4,6a,6b,8a,11,12,14b-heptamethyl-14-oxo-1,2,3,4,4a ,5,6,6a,6b,7,8,8a,9,10,11,12,12a,14,14a,14b- icosahydropicene-4-carboxylate
C6D		(1(2(trifluoromethyl)phenyl)1H1,2,3triazol4yl) methyl(3R,4R,6aR,6bS,8aR,11R,12S,12aR,14bS) 3acetoxy4,6a,6b,8a,11,12,14bheptamethyl14oxo1 ,2,3,4,4a,5,6,6a,6b,7,8,8a,9,10,11,12,12a,14, 14a,14b-icosahydropicene-4-carboxylate
C6E		(1-(4-methoxyphenyl)-1H-1,2,3-triazol-4-yl) methyl (3R,4R,6aR,6bS,8aR,11R,12S,12aR,14bS) -3-acetoxy-4,6a,6b,8a,11,12,14b-heptamethyl- 14-oxo-1,2,3,4,4a,5,6,6a,6b,7,8,8a,9,10,11, 12,12a,14,14a,14b-icosahydropicene -4-carboxylate
C6F		(1-(3-(trifluoromethyl) phenyl)-1H-1,2,3-triazol-4-yl) methyl (3R,4R,6aR,6bS,8aR,11R,12S,12aR,14bS) -3-acetoxy-4,6a,6b,8a,11,12,14b-heptamethyl- 14-oxo-1,2,3,4,4a,5,6,6a,6b,7,8,8a,9,10,11, 12,12a,14,14a,14b-icosahydropicene -4-carboxylate
C6G		(1-(3-bromophenyl)-1H-1,2,3-triazol-4-yl) methyl (3R,4R,6aR,6bS,8aR,11R,12S,12aR,14bS) -3-acetoxy-4,6a,6b,8a,11,12,14b-heptamethyl- 14-oxo-1,2,3,4,4a,5,6,6a,6b,7,8,8a,9,10,11,12, 12a,14,14a,14b-icosahydropicene-4-carboxylate
C6H		(1-(4-bromophenyl)-1H-1,2,3-triazol-4-yl) methyl (3R,4R,6aR,6bS,8aR,11R,12S,12aR,14bS) -3-acetoxy-4,6a,6b,8a,11,12,14b-heptamethyl- 14-oxo-1,2,3,4,4a,5,6,6a,6b,7,8,8a,9,10,11,12, 12a,14,14a,14b-icosahydropicene-4-carboxylate
C6I		(1-(4-chlorophenyl)-1H-1,2,3-triazol-4-yl)methyl (3R,4R,6aR,6bS,8aR,11R,12S,12aR,14bS)-3-acetoxy -4,6a,6b,8a,11,12,14b-heptamethyl-14-oxo-1,2,3, 4,4a,5,6,6a,6b,7,8,8a,9,10,11,12,12a,14,14a,14b- icosahydropicene-4-carboxylate
C6J		(1-(4-fluorophenyl)-1H-1,2,3-triazol-4-yl) methyl (3R,4R,6aR,6bS,8aR,11R,12S,12aR,14bS)-3-acetoxy -4,6a,6b,8a,11,12,14b-heptamethyl-14-oxo-1,2,3,4,4a, 5,6,6a,6b,7,8,8a,9,10,11,12,12a,14,14a,14b -icosahydropicene-4-carboxylate
C6K		(1-(4-(trifluoromethyl) phenyl)-1H-1,2,3-triazol-4-yl) methyl (3R,4R,6aR,6bS,8aR,11R,12S,12aR,14bS) -3-acetoxy-4,6a,6b,8a,11,12,14b-heptamethyl- 14-oxo-1,2,3,4,4a,5,6,6a,6b,7,8,8a,9,10,11, 12,12a,14,14a,14b-icosahydropicene- 4-carboxylate

### Analysis of binding interactions at RBD of spike protein

**Figure 4:**
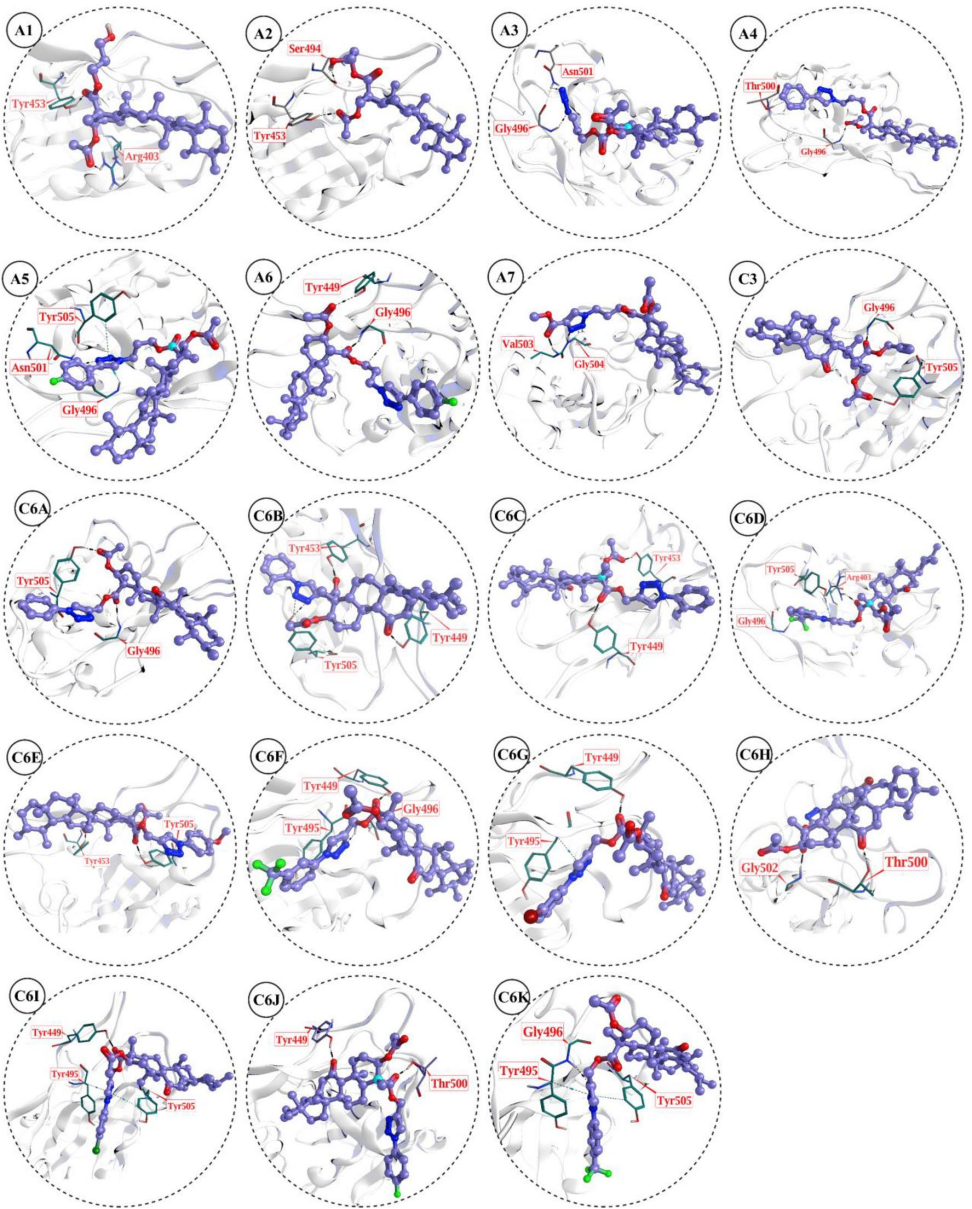
3D molecular docking interaction of selected compounds against spike protein receptor binding domain PDB ID: 6MOJ.

The binding modes of 19 AKBA derivatives were analyzed which showed that these molecules exhibit good scores and mediate good docking interaction. The AKBA docking score at spike protein receptor binding domain is − 5.8732 kcal/mol and mediates three hydrogen bonds with TYR505, TYR473 and GLN409 of RBD at 2.06 Å, 1.14 Å and 3.09 Å, respectively. Among those selected compounds, **C-6C** exhibited the highest docking score of − 5.73 kcal/mol, followed by **C-6F, C6F, C3, A6, C6E, C6D, C6B, A5, A1, A4, C6A, A7, A3,** and **A2** which have docking score in range of − 5.55 to − 4.73 kcal/mol. The compound **A1** interacts with residues of spike protein TYR453 and ARG403 through hydrogen bond, while **A2** mediates H-bonds with SER494 and TYR453 to be stabilized at RBD interface. The compounds **A3** and **A4** make two hydrogen bonds with GLY496 and ASN501, and GLY496 and THR500, respectively. While **A5** forms multiple H-bonds and hydrophobic (π-H) interactions with ASN501, GLY496, and TYR505. Similarly, **A6** mediates numerous hydrogen bonds with GLY496 (2X) and TYR449 to stabilize its binding. The compounds **A7** and **C3** bind with GLY504 and VAL503, and GLY496 and TYR505, respectively by two hydrogens that firms the ligand in the active pocket. Similarly, **C6A** is attached at the RBD by mediating two H-bonds with GLY496 and TYR505. Moreover, **C6B** also forms multiple H-bonds with TYR453, TYR505 (2X), and TYR449, and **C-6C** forms two H-bonds with TYR449 and TYR453 which holds the ligand firmly. While **C6D** makes two H-bonds with ARG403 and GLY496 and a π-H bond with TYR505, and **C6E** forms two H-bonds with TYR505 and TYR453. Likewise, **C6F** makes a π-H bond with TYR495 and two H-bonds with TYR449 and GLY496 indicating ligand’s firmness in the protein complex. Whereas **C6G** makes an H-bond and a π-H bond with TYR 449 and TYR495, respectively, and **C6H** makes two H-bonds with GLY502 and THR500. The compound **C6I** interacts with spike protein through two π-H bonds with TYR449 and TYR495, and a H-bond with TYR505, while **C6J** interacts with THR500 and TYR449 through H-bonds. Lastly, the compound **C6K** also makes two π-H bonds with TYR495, TYR505 and a H-bond with GLY496. The docking predicted interaction of these compounds at molecular level, reflecting firm binding of compounds at RBD interface (Table S1 and Fig. 4).

### Pharmacokinetics and drug-likeness of selected compounds

For the drug’s dose, availability at the predicted region of action, and bonding to the target of therapeutic concern to possess pharmacokinetic and drug-like qualities^44^. Therefore, the pharmacokinetic and drug-like characteristics were estimated for the selected inhibitors ligand against spike protein (RBD). The selected compounds have molecular weights (MW) ranging from 556.82 to 748.79 g/mol. All these inhibitor compounds possess the number of hydrogen bond acceptors (NHBAs) < 10, and the number of hydrogen bond donors (NHBDs) < 5. Also, the number of rotatable bonds (NRB) is less than 10 for the chosen compounds (Tables 2 and S2). The predicted polarity and topological surface area (TPSA) are in an acceptable range from 46.53 to 100.38 Å^2^ (a molecule with a TPSA value less than 140 Å^2^ has a high probability of good absorption or oral bioavailability). The LogP o/w (octanol–water partition coefficient) value is up to 5, and the obtained LogP score of compounds indicated good absorbency or lipophilicity (LogS) score ranging from − 7.75 to − 9.96 showed the solubility of molecules. All the compounds obey Lipinski's rule of five. It is pertinent to mention that a compound violating any Lipinski rule results in only molecular weight (MW) > 500 Da. Although Lipinski’s Rule of Five is a helpful guideline, it should be noted that it is not infallible and that many well-marketed medications do not meet all its requirements^62,63^. Indeed, molecules with molecular weights above 500 Da may still be deemed drug-like provided they possess additional desirable characteristics, making the molecular weight criterion a flexible one^64,65^. Natural compounds such as those derived from plants, marine life, and microorganisms can have greater molecular weights than synthetic ones and can be employed as drugs or therapeutic agents. Antibiotics, anti-cancer drugs, and immunosuppressants drugs are a few examples^66–68^. Another crucial factor in determining oral bioavailability is molar refractivity (MR), defined by the Ghose rule ^69^, and its value, as per the Lipinski rule^62^, should range from 40 to 130 for fulfilling the drug-likeness criteria by all compounds except **A2, A6**, and **C-6H**. Besides, Ghose, Veber^70^, Egan^71^, and Muegge^72^ rules were used to examine the drug-likeness of compounds. The compounds fulfill the rule of Veber and Muegge while the violation is shown by some compounds in the Egan rule (WLOGP > 500) by **A2, A5, C6F**, and **C6G**. The pharmacokinetic evaluation showed that these compounds have good absorption in the gastrointestinal tract (GIA) except **A4, A5, A6, C6A, C6F**, and **C6G**. None of the compounds can cross the blood–brain barrier (BBBP). Except for **A3**, and **A7** the other compounds do not display features typical of P-glycoprotein substrates. None of the compounds showed inhibitory potential for cytochrome p450 enzymes, CYP1A2 CYP2C9, and CYP2C19, except **A4, A6, A7**, **C6G**, and **C-6D** which show inhibition for CYP2D6. Besides these, all the compounds are non-allergic as predicted by CHAIPred and cannot elicit any kinds of immune response. The toxicity analysis as predicted by server ProTox-II for the selected compounds indicates that these compounds are non-toxic except the **A4, A5, A6, C6F**, and **C6G** which show immunotoxicity and only A5 shows cytotoxicity. The computed LD50 values range from 500 to 3300 mg/Kg and most of the ligands fall in predicted toxicity class IV, while compounds **A6** and **C3** fall in predicted toxicity class V. The pharmacokinetic features of these candidates are encouraging, and they may prove useful as inhibitors in future clinical studies specially **A3, A4, C3, C6A, C6B, C6C, C6E, C6H, C6I** and **C6J** which can not violate any rules of Lipinski’s, pharmacokinetics and drugs-like (Tables S3 and S4).

**Table 2:** Physiochemical properties and allergenicity of AKBA and its selected derivatives against Spike protein receptor binding domain PDB ID (6MOJ) using server SwissADME and server CHAIPred.

Names	Physiochemical properties					Lipophilicity	Water solubility	Allergenicity CHAIPred
	MW (g/mol)	HBA	HBD	NRB	TPSA (Å^2^)	Log/*P*o/w	Log/ESOL	
**AKBA**	546.82	3	1	8	46.53	5.54	− 9.43	Non-allergen
**A1**	556.82	5	1	7	72.83	5.11	− 8.05	Non-allergen
**A2**	556.82	5	1	7	72.83	5.11	− 8.05	Non-allergen
**A3**	581.83	7	0	8	102.35	5.52	− 9.14	Non-allergen
**A4**	683.96	6	0	9	83.31	4.04	− 9.97	Non-allergen
**A5**	701.95	7	0	9	83.31	4.15	− 10.14	Non-allergen
**A6**	701.95	7	0	9	83.31	3.15	− 10.14	Non-allergen
**A7**	680.94	8	0	9	109.61	5.56	− 9.27	Non-allergen
**C3**	550.77	5	0	5	69.67	4.97	− 7.75	Non-allergen
**C6A**	669.89	7	0	7	100.38	5.34	− 9.05	Non-allergen
**C6B**	683.92	7	0	7	100.38	5.83	− 9.37	Non-allergen
**C6C**	683.92	7	0	7	100.38	5.83	− 9.37	Non-allergen
**C6D**	683.92	7	0	7	100.38	5.83	− 9.37	Non-allergen
**C6E**	737.89	10	0	8	100.38	5.79	− 9.96	Non-allergen
**C6F**	699.92	8	0	8	109.61	5.89	− 9.15	Non-allergen
**C6G**	748.79	7	0	7	100.38	5.71	− 9.98	Non-allergen
**C6H**	748.79	7	0	7	100.38	5.64	− 9.98	Non-allergen
**C6I**	704.34	7	0	7	100.38	5.45	− 9.66	Non-allergen
**C6J**	687.88	8	0	7	100.38	5.32	− 9.22	Non-allergen
**C6K**	737.89	10	0	8	100.38	5.95	− 9.96	Non-allergen

MW, molecular weight; TPSA, topological polar surface area; NRB ,number of rotatable bonds; HBA, hydrogen bond acceptor; HBD, hydrogen bond donor; Log Po/w,  partition coefficient octanol/water; Log/ESOL, estimated solubility.

### In-vitro validation of our computational results

**Figure 5:**
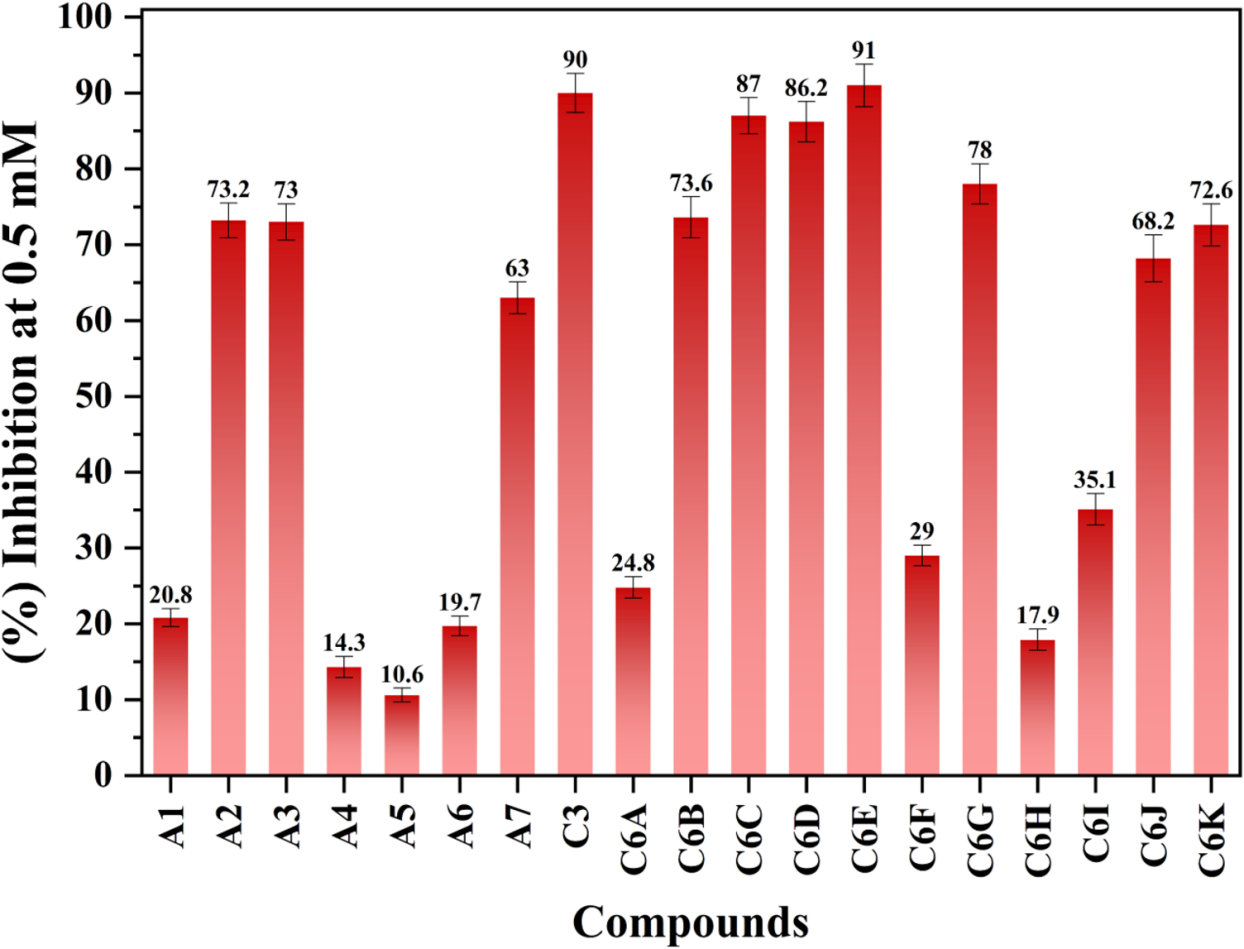
Percent inhibition of selected compounds against SARS CoV-2 spike protein RBD.

Furthermore, the inhibitory mechanism of the selected AKBA derivatives was evaluated activity against SARS-CoV-2 spike protein. Among the tested hits, eleven compounds displayed > 50% inhibition of RBD (in the range of 73.00–91.00% inhibition) while rest eight compounds exhibited < 50% inhibition and were declared inactive. The basic skeleton of these compounds is similar while the R group is varied. For instance, the invitro activity demonstrates that compound **A1** with methanol substitution is inactive, in contrast, **A2** and **A3** with methyl 4-methyl benzenesulfonate and, azido methane substitutions, respectively exhibited good inhibitory capability with 73.20% and 73.00% inhibition respectively. While inhibitory potency of **A7** was somehow declined (63.00% inhibition) with the substitution of methyl 1-methyl-1H-1,2,3-triazole-4-carboxylate as compared to **A2** and **A3**. Similarly, compound **C-3** with prop-1-yne substituent resulted in high inhibitory potential with 90.00% inhibition, however, **C6A** exhibited < 50% inhibition and was found inactive. On the other hand, **C6B** with the substitution of 4-methyl-1-(o-tolyl)-1H-1,2,3-triazole exhibited good inhibitory capability against SARS CoV-2 spike protein with 73.60% inhibition. The inhibitory potency of **C6C** and **C6D** was enhanced with the substitution of 1-(2-methoxyphenyl)-4-methyl-1H-1,2,3-triazole and 4-methyl-1-(2-(trifluoromethyl)phenyl)-1H-1,2,3-triazole, resulted in 86.20% inhibition of both compounds as compared to **C6B**. Interestingly, the substitution of 1-(4-methoxyphenyl)-4-methyl-1H-1,2,3-triazole in **C3** and C**6E** further increased its potency against RBD and was found to be the most active molecule with 91.00% inhibition. We observe that compounds **C6F**, **C6H** and **C6I** has < 50% inhibition, while **C6G** with 1-(3-bromophenyl)-4-methyl-1H-1,2,3-triazole substitution significantly inhibited the protein with 78.00% inhibition. However, the inhibitory effect of **C6J** declined with the substitution of 1-(4-fluorophenyl)-4-methyl-1H-1,2,3-triazole (68.20% inhibition). While **C6k** with 4-methyl-1-(4-(trifluoromethyl) phenyl)-1H-1,2,3-triazole substitution exhibited 72.6% inhibition (Fig. 5).

### Molecular dynamic simulation

#### Protein stability analysis (RMSD)

**Figure 6:**
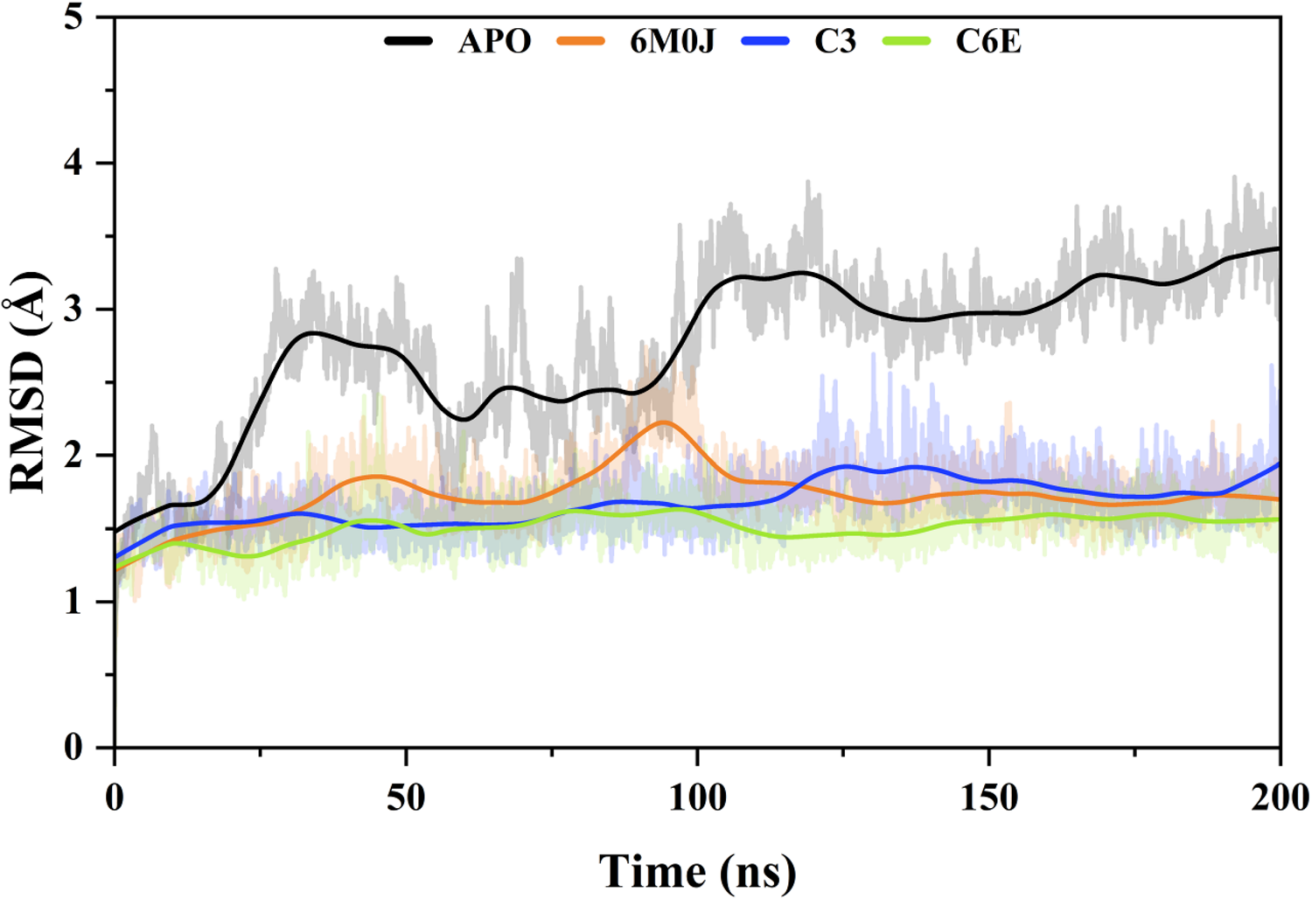
Root mean square deviation analysis of spike protein RBD domain in complex with human ACE2 receptor (6M0J), free state (APO) and selectd compounds (C3 and C6E) in 200 ns simulation time.

To investigate the structural modifications that occur in proteins because of the binding of inhibitors (compounds) we used AMBER22 to simulate the interaction that would occur between Spike protein RBD and the compounds that were chosen. The topology and coordinate data were produced with the help of the LEap modules in AMBER22, which utilized the ff198SB forcefield. Based on good inhibition against spike protein RBD we select two compounds **C3** and **C6E** for simulation to further confirm the docking interaction. The root-mean-square deviation (RMSD) was computed to gain an understanding of the degree to which the compounds diverged from their primary structure. The total average RMSD for APO was 2.7429 ± 0.0512 Å. A fluctuation of 2.8345 Å was observed at the start of the simulation at 33 ns and then 3.2441 Å at 107 ns, after that the protein was stable throughout the entire simulation. For the spike protein 6MOJ, the total RMSD value is 2.7429 ± 0.023 Å. The protein was stable through the entire simulation while a small rise was observed from 85 to 95 ns of 2.9248 Å. Similarly, the total average RMSD value for the ligand **C3** was calculated at 1.6774 ± 0.012 Å. A small fluctuation of 1.9065 Å was observed at 127 ns then the entire simulation the ligand was stable. The RMSD value for ligand **C6E** was 1.5083 ± 0.0132 Å, however small variation was observed at 44 ns (1.5561 Å), 80 ns (1.6187 Å), and 98 ns (1.6205 Å) than stable at the end of the simulation (Fig. 6).

#### Residues fluctuations analysis (RMSF)

The root mean square fluctuation (RMSF) is a measurement performed to assess the extent of deviation of an atomic position relative to its average position within the framework of a protein structure. In this study, the RMSF analysis was performed on the Spike protein–protein complex with specific compounds (**C3** and **C6E**) to investigate the fluctuations of these compounds concerning the protein. Increased RMSF values after compound attachment suggested a more flexible structure, which indicated well for subsequent interactions with ligand molecules. The average RMSF value for protein 6MOJ was calculated 1.0431 ± 0.012 Å. The protein was stable at the end of the simulation while some fluctuation was observed at 38 ns (3.4612 Å), 53 ns (2.6399 Å), and 187 ns (3.1878 Å). Similarly, for the APO form of protein, the average RMSF value was 0.9697 ± 0.065 Å. The residue fluctuation was observed at the start of the simulation at 35 ns (1.3634 Å) and 145 ns (2.3890 Å) for the APO form of spike protein RBD. The average RMSF value of inhibitor compound **C3** was calculated as 0.93210 ± 065 Å. The compound was stable throughout the simulation while small residual fluctuation was detected at 38 ns of 2.4194 Å and 2.3456 Å at 146 ns. For the ligand **C6E,** a small residual increase was observed at 38 ns (2.1943 Å), 143 ns (1.9673 Å), and 187 ns (1.8934 Å) then stable at the end of the simulation with an average RMSF value of 0.9537 ± 065 Å (Fig. S1).

#### Radius of gyration (Rog) analysis

**Figure 7:**
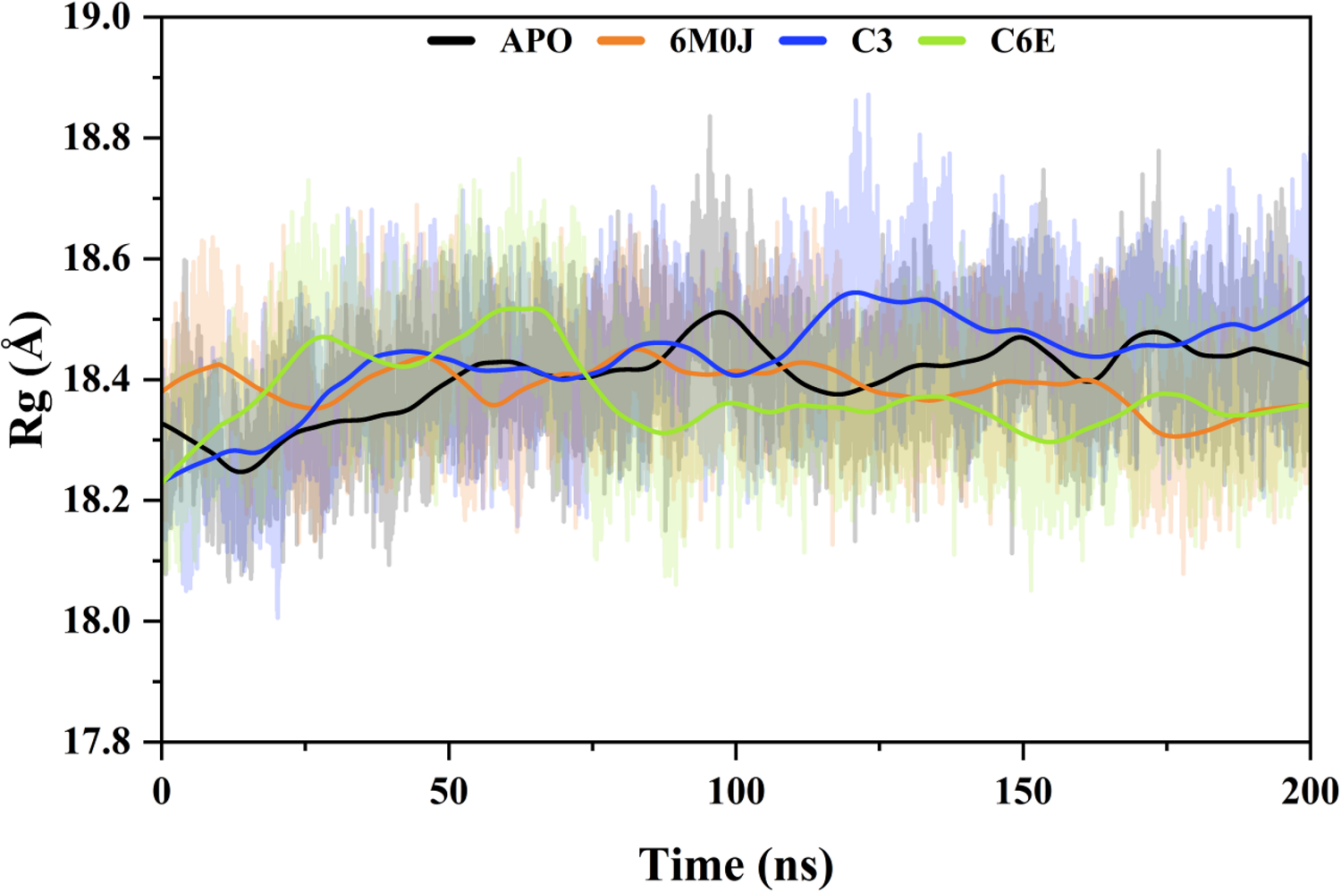
Radius of gyration analysis was used to calculate the compactness of the spike RBD domain in free (APO), activated state (6M0J) and selected inhibitors attached (C3 and C6E).

It is important in figuring out the radius of gyration because it gives a basic overview of an object's shape and helps forecast its behaviors under different conditions, considering things like stability, potential for energy transfer and preservation, and responsiveness to outside influences. The average Rog value for the APO form of protein is 18.4030 ± 0346 Å. A small increase was observed from the start of the simulation till 98 ns (18.5063 Å) then remained stable at the end of the simulation. Similarly, the average Rog value for spike protein 6MOJ was calculated 18.3886 ± 3.981 Å and during simulation small fluctuation was detected at 48 ns (18.4274 Å) and 83–89 ns (18.4467 Å). For the inhibitor compound **C3** the total Rog value was calculated as 18.4347 ± 0321 Å. The compound was stable while showing an increase of 43 ns (18.4467 Å) to 120 n (18.5437 Å) at the start of the simulation. Similarly, the ligand **C6E** shows an average Rog value of 18.3742 ± 0321 Å. At the start of the simulation, an increase in Rog value was observed from 25 to 65 ns (18.5073 Å) then became stable at the end of the simulation (Fig. 7).

### Spike protein dominant motions

**Figure 8:**
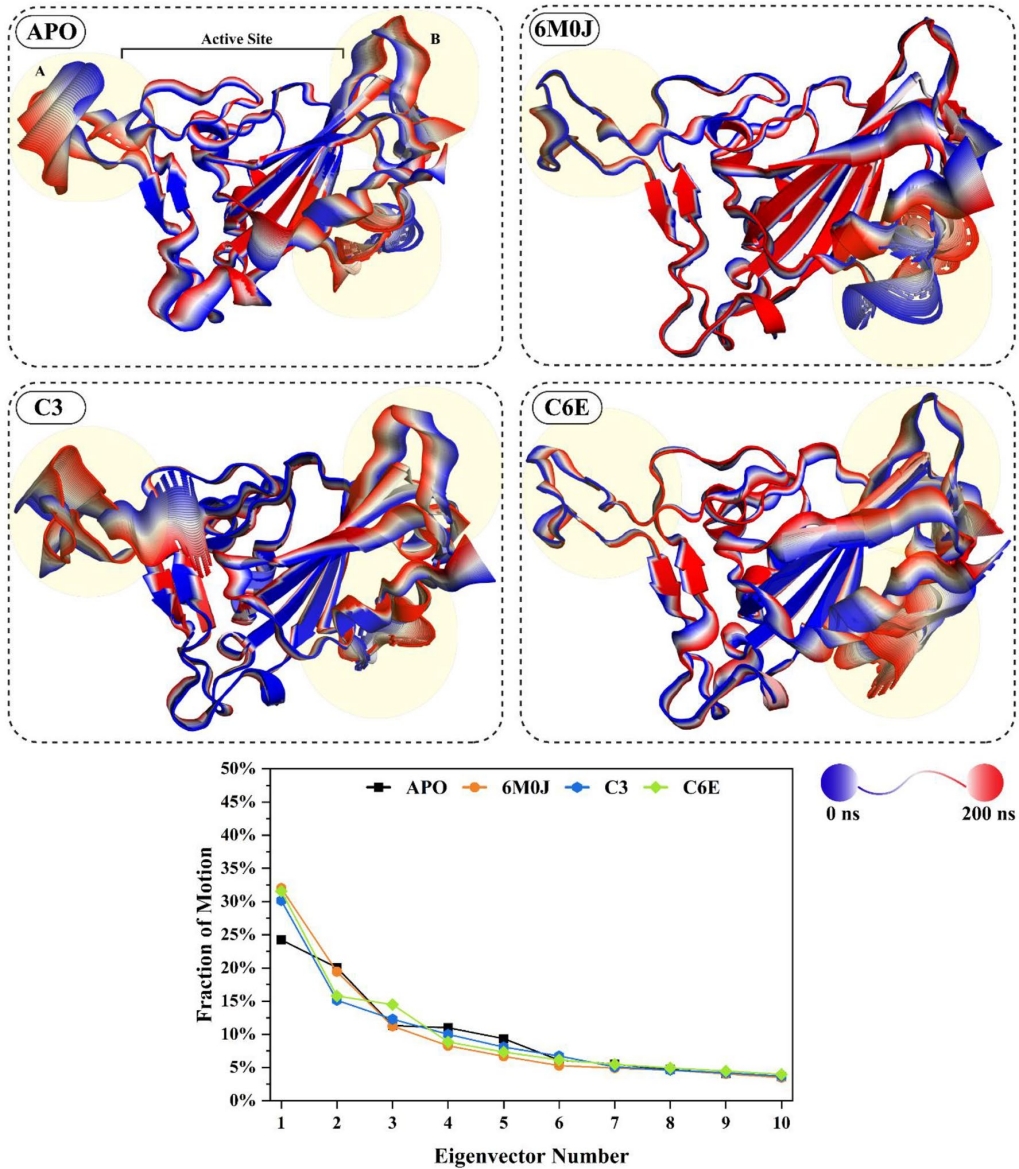
Principal component analysis (PCA) was conducted on four distinct systems which include the spike protein in its free state (APO) as the reference, the activated 6M0J system (comprising the spike RBD domain and ACE2 complex), and two systems with selected inhibitors attached (C3 and C-6E). The cumulative movements derived from the top ten eigenvectors for each of these systems are expressed as percentages in the 2D graph. The color-coded cartoon representation transitions from blue to red and shows the regions of the protein from their initial state to their altered state at the 200 ns simulation period. The residues region where the dominant motions were observed were highlighted in yellow.

The principal component analysis (PCA) was used to calculate the dominant motions in the spike protein based on the Cα atoms in a 200 ns simulation trajectory. The total mobility observed in the protein was calculated by the eigenvalues, for which the eigenvectors were calculated from the covariance matrix. Ten eigenvectors for each system were calculated to explore the dominant motions that contribute to the significant structural changes during simulation (Fig. 8). The highest motions observed were in the reference-activated system 6M0J with a value of 32% in 200 ns simulation, followed by the **C6E** system (31%). The motions observed in the protein were in the protein–protein interaction interface, where the loop B side shows significant structural modification (Fig. 7). The internal sheets of the structure went through structural confirmation upon binding with the *h*ACE2 protein. Meanwhile, the **C6E** system shows slight confirmation of the A loop position, and the B loop region shows most of the motions in the cartoon representation. Comparing the motions in the active state (6M0J) and inhibited system **(C6E),** they were opposite in direction, whereas the internal sheets in the C6E system do not show any structural modification (stays blue). The **C3** system also shows a variation in the structural dynamic of 30% from its initial coordinates in the simulation run. The **C3** system cartoon representation from the 200 ns simulation shows that the active site region of the protein depicts most of the structural confirmation of the **C3** protein. The APO system (spike in free state) shows the lowest structural confirmation during the 200 ns simulation run (24%). The APO protein cartoon representation from the PCA analysis shows that the spike structure shows most of the motions in the overall loop’s regions of the protein. The PCA results from the 200 ns simulation of the spike protein's dynamics in free activated and inhibited states highlight key insights into structural mobility and interactions. The most significant movements were observed in the reference-activated system (6M0J) and the **C6E** system, particularly at the protein–protein interaction interfaces and loop regions. Notably, the directional opposition of movements between the active (6M0J) and inhibited **(C6E)** states emphasizes the insightful impact of ligand binding on the protein’s conformation. These results represent valuable perspectives of the selected compounds for therapeutic design to emphasize the importance of targeting areas of high mobility and conformational changes within the spike protein to disrupt its function effectively.

**Figure 9:**
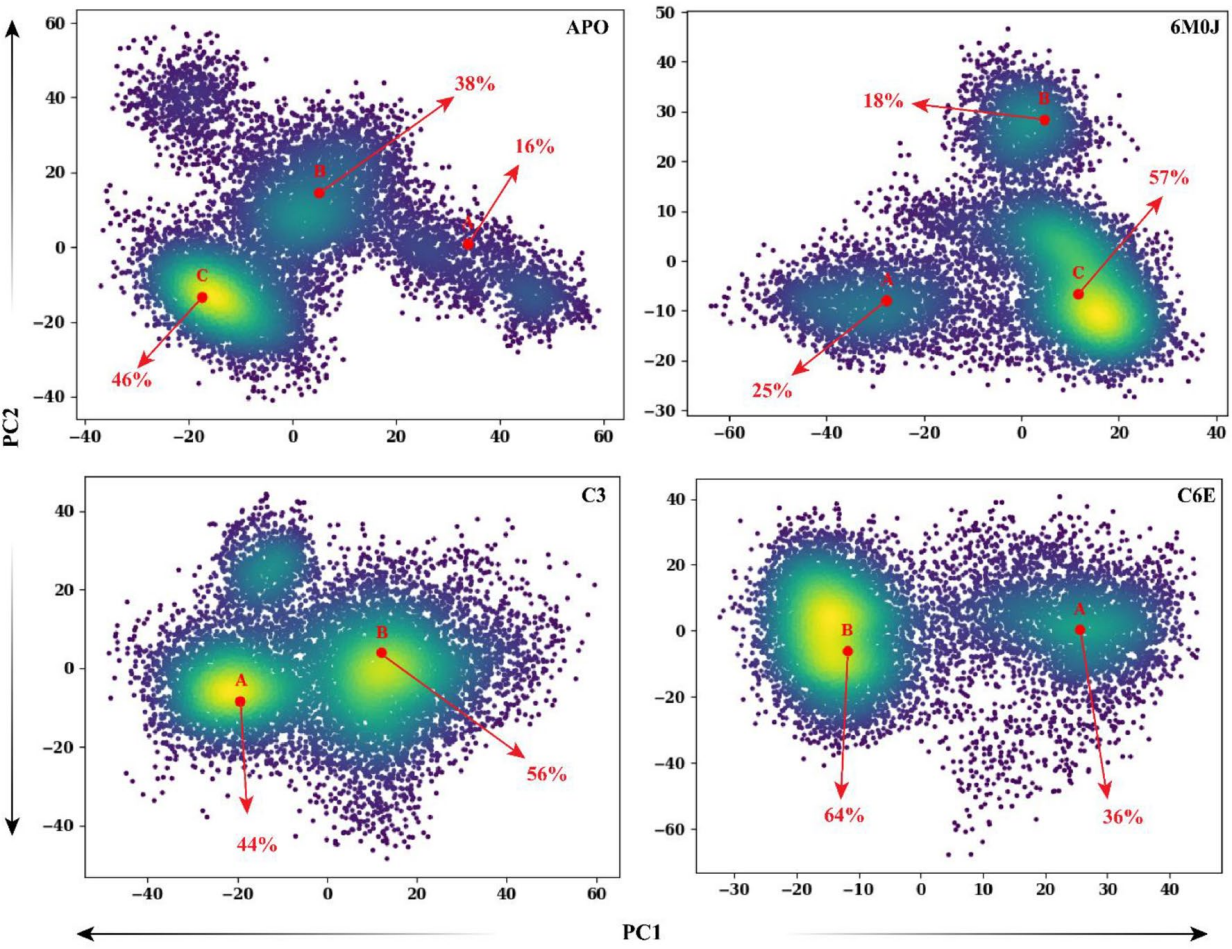
Flipping over the orientation of PC1 and PC2 components in the PCA analysis for the spike RBD domain in the free state (APO), activated state (6M0J), and systems with attached selected inhibitors (C3 and C6E) was conducted. Color coding was utilized to represent the density of frames over the simulation from blue, green, and yellow, where yellow means high density. The clusters within each system were labeled A, B, and C, and the simulation duration for which the system remained in each cluster was represented as a percentage.

The spike RBD domain underwent structural conformation changes in the free state, activated and inhibited during the simulation run. To observe these changes, the PC1 and PC2 values were plotted against each other. The transitions of the protein during the simulation run and where it stays the most, at which confirmation was shown in the Fig. 9. Three distinct confirmation clusters were observed in the APO system. Cluster A shows that the system stayed for 16% of the simulation time, from which the system moves to cluster B, where the system shows a diverse behavior for 38% of the simulation time. The system stabilizes in the cluster C region, where the simulation ends and the overall simulation time stayed in cluster C was 46%. The activated reference system 6M0J shows rapid conformational changes in the clusters. Cluster A shows that the simulation stayed at 25% and then shifted to cluster B with a stay of 18% simulation time. The system stabilizes in cluster C, where the simulation stays for the rest of the simulation period (57%). The behavior of the inhibitor-attached systems changed due to the inhibitor attachment with the protein active site. The **C3** system shows two dominant clusters, where the system stayed in cluster A for 44% of the simulation time and then shifted to cluster B (56%) where the system stayed till the end of the simulation time. Similarly, the **C6E** system shows two distinct structural confirmations, where the system stays at cluster A for 36% and then stabilizes in cluster B (64%) for the rest of the simulation time. The structural conformation changes based on PC1 and PC2 of the spike RBD domain in the free state (APO), activated state (6M0J), and selected inhibitor attached states (C3 and C6E) revealed notable differences. In the APO system, a progression through three conformation clusters was observed, with a significant duration in the final cluster, while the activated 6M0J system showed quicker conformational transitions, ultimately stabilizing in its final cluster. In contrast, the inhibitor-attached systems (**C3** and **C6E**) displayed distinct patterns from the reference Apo and 6M0J, predominantly occupying two major clusters each, reflecting the influence of inhibitor attachment on the protein’s conformational dynamics.

**Figure 10:**
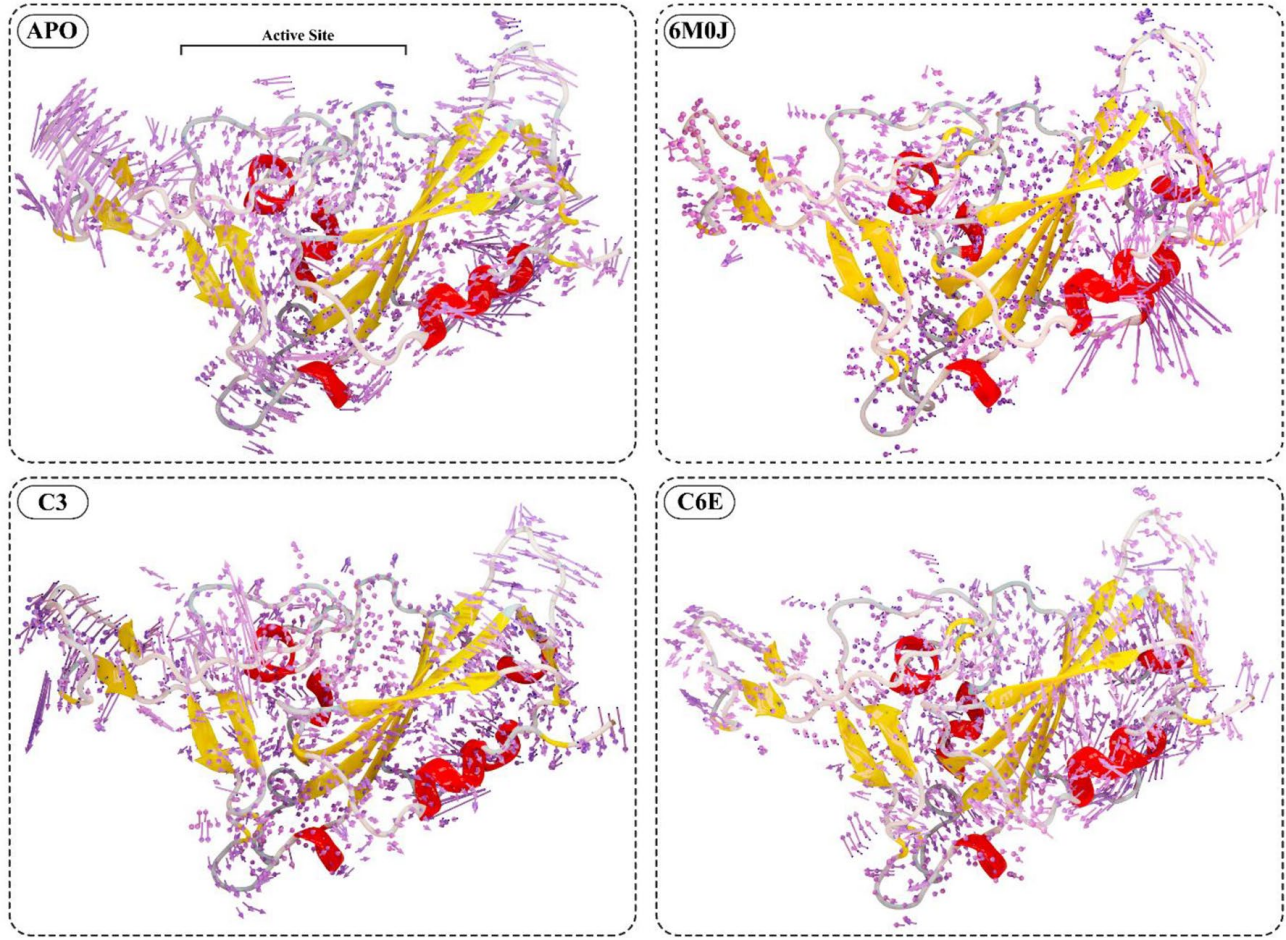
The porcupine plot, derived from the PCA analysis which includes the spike RBD in free state (APO), activated state (6M0J), and selected inhibitors attached systems (C3 and C6E). The arrow size indicates the extent of movement, and the arrow's orientation reveals the direction of the motion.

To further explore the direction and magnitude of the principal movements, porcupine plots were constructed for each system (Fig. 10). In the porcupine plot, the arrow length shows the magnitude of the motion caused in the residue positions, while the arrowhead shows the direction of the movement. The free state spike protein (APO) shows a close confirmation in the porcupine plot, where the active site residues move toward the active site region. In contrast, the magnitude and direction of the movements change in the active state of the 6M0J system. The active pocket’s right side moves towards the active site while affecting the helixes in the vicinity of the active pocket, while the left side remains stable with minor motion deviation. Comparing the APO and activated state 6M0J with the **C3** inhibited state, the behavior of movement in the inhibited system was changed. The active pocket of the **C3** inhibitor attached system moves outward, which retains an open conformation of the spike RBD. Similarly, the **C6E** follows the pattern of the **C3** system, where the active pocket moves away from the center of the active site. The **C3** system shows a higher magnitude of movement when compared with the **C6E** system. The outcomes from the porcupine plot results show distinct movement patterns of spike RBD protein in free, active, and inhibited states. In the free state spike RBD (APO), active site residues converged towards the center of the active site, which indicates a compact conformation, while the active state (6M0J) displayed divergent movement, having one side of the active pocket shifting towards the active site and affecting the helices in the active pocket vicinity. In contrast, inhibitor-attached systems (**C3** and **C6E**) showed an outward movement of the active pocket, suggesting an open conformation, where the **C3** system particularly exhibited a more noticeable movement magnitude than **C6E**, which highlights the variable impact of the selected inhibitors on the protein’s structure.

### Hydrogen bond analysis

Hydrogen bonds are essential in maintaining the structure and function of biological molecules. In molecular dynamic simulations, they play a crucial role in determining the dynamics and stability of molecular systems. The SARS-CoV-2 spike protein interacting residues with the ACE2 receptor include ASN155, GLN166, GLY170, GLY164, GLN161, TYR117, THR168, GLY171, ASN155, TYR117, GLY114, LYS85, and TYR121. The bond lifetime between the ligands and these residues ranged from 11.00 to 81.20%. The ligand **C3** makes hydrogen bond with residue GLY-170, GLY164, GLN161 and ASN169 which have a bond occupancy of 71.95%, 42.30%, 16.80% and 15.90%. The supporting residues GLN161 (5X), TYR117 (4X), GLN166 (4X), ARG71 (2X), TYR173, THR168, and TYR121 which have a bond occupancy > 10% make connection with ligand. Similarly, the ligand atom **C6E** makes hydrogen bonds with receptor residues THR138, GLN166 (2X), SER-62, THR138 (2X), TYR19 with a bond occupancy of 65.24%, 39.00%, 33.00%, 31.00%, 24.00%, 20.30% and 17.00% respectively. The supporting residues THR168, TYR19 (4X), GLN-66 (2X), THR138 (2X), ASN118 (5X), ARG14(2X), GLN161, GLY170 and GLU152(2X) which make hydrogen bond with receptor atom having bond occupancy > 10%. The investigation of hydrogen bond interactions revealed that the chosen chemicals establish strong bonds with the key residues present in the active pocket of Spike protein (Table S5).

### Binding free energy calculation

The Molecular Mechanics and Generalized Born Solvent Accessible Surface Area (MM-GBSA) technique has been utilized to determine the binding free energies between various inhibitor chemicals and Spike protein (6MOJ). Because it can provide information about the strength of a protein–ligand interaction, the use of estimated binding free energy is important in the field of drug design. This in turn makes it easier to assess potential chemicals' binding affinities. In this study, we examined several types of energies, including van der Waals forces (ΔE_VDW_), electrostatic energy (ΔE_EEL_), polar solvation energy (ΔE_GB_), the nonpolar component of solvation energy (ΔE_SURF_), and the total binding free energy (ΔG_TOTAL_) for inhibitors compounds with SARS CoV-2 Spike protein (RBD) (Table 3). The total binding free energy of protein 6MOJ is (ΔG_TOTAL_) − 64.42 ± 0.19 kcal/mol. The reduced surface area of protein is − 13.47 Å. The total (ΔE_VDW_) energy is − 92.32 ± 0.14  kcal/mol, total (ΔE_EEL_) − 627.31 ± 1.0 kcal/mol, and total (ΔE_GB_) is 668.68 ± 0.94 kcal/mol. The total binding free energy for compound **C3** is (ΔG_TOTAL_) − 38.0 ± 0.08 kcal/mol with a reduced surface area of − 5.60 Å. The total (ΔE_VDW_) is − 41.20 ± 0.12 kcal/mol, (ΔE_EEL_) is − 9.90 ± 0.09 kcal/mol and (ΔE_GB_) 18.68 ± 0.09 kcal/mol. Similarly, for the ligand compound **C6E,** the total binding free energy (ΔG_TOTAL_) is − 41.98 ± 0.08 kcal/mol with a reduced surface area of − 6.55 Å. For the ligand **C6E** the (ΔE_VDW_) is − 37.75 ± 0.11 kcal/mol, (ΔE_EEL_) is − 15.85 ± 0.10 kcal/mol and (ΔE_GB_) is 18.17 ± 0.12 kcal/mol.

**Table 3:** Binding free energy of the simulated inhibitor compounds C3 and C6E.

Complex	MM-GBSA calculations (Unit’s kcal/mol) Differences (Receptor–Ligand–Complex)				
	ΔE_VDW_	ΔE_EEL_	ΔE_GB_	ΔE_SURF_	ΔG_TOTAL_
6M0J	− 92.32 ± 0.14	− 627.31 ± 1.0	668.68 ± 0.94	− 13.47 ± 0.01	− 64.42 ± 0.19
C3	− 41.20 ± 0.12	− 9.90 ± 0.09	18.68 ± 0.09	− 5.60 ± 0.01	− 38.0 ± 0.08
C6E	− 37.75 ± 0.11	− 15.85 ± 0.10	18.17 ± 0.12	− 6.55 ± 0.17	− 41.98 ± 0.08

ΔE_EEL_, electrostatic energy; ΔE_VDW_, van der Waals energy; ΔG_TOTAL_, total binding free energy; ΔE_GB_, polar solvation energy; ΔE_SURF_, the nonpolar component of the solvation energy.

## Discussion

The widespread infectivity of the COVID-19 pandemic has made it a serious public health concern^73^. Human lung epithelial cells are infected when the SARS-CoV-2 virus attaches its spike protein to the human ACE2 receptor on the cell surface^74^. After binding to the *h*ACE2 receptor the SARS-CoV-2 and other coronaviruses, allow their RNA to penetrate the target cell^75^. The estimated binding affinity between SARS-CoV-2 and *h*ACE2 is ~ 16 nM which is as much as twenty times higher than that of SARS-CoV^76^. To prevent virus entry into human cells, scientists are working to create drug compounds that interfere with the spike protein’s ability to connect to *h*ACE2^77,78^. Several investigations towards blocking SARS CoV-2 entry into the host cell have focused on the spike protein^79–83^. Also, vaccines have been developed as an effective agents but still possess some modest side effects^84–86^. Over time, viruses can undergo mutations that lead to the formation of new versions that can either fully or partially prevent immunization and develop resilience to current medications and vaccines. The creation of novel inhibitors may offer substitute therapeutic approaches that are successful in combating newly developing variations. Designing inhibitors against the spike protein could aim for a broad-spectrum effect, targeting multiple variants or even different coronaviruses. Since drugs developed to treat SARS-CoV-2 could provide an effective first line of defense against future coronaviruses, their development is more rational and preferable^77^. Similarly, in-silico methods are becoming increasingly significant in the pharmaceutical industry^87^. In-silico medication design affects the overall drug development schedule by facilitating the quick identification and discovery of innovative therapeutic medicines^88–91^. Herein, we employed a combination of computational methods like structure-based virtual screening and 2D-similarity searching and an enzyme inhibition bioassay to uncover novel potential inhibitors of SARS-CoV-2 spike protein.

In this study, the crystal structure of the spike protein receptor binding domain (RBD) was taken together with the human *h*ACE2 receptor.The natural products and their derived compounds were targeted against spike protein through virtual screening. Among the top 10% natural compounds, the acetyl 11-keto-β-boswellic acid (AKBA) showed good binding and docking score which was further confirmed by in-vitro inhibition bioassay where AKBA showed > 80% inhibition of spike protein RBD. Based on in-vitro results of AKBA, we searched for more AKBA-like molecules in our in-house database through 2D-similarity searching. Later, we obtained 19 compounds that have 85% similarity with AKBA with docking scores of − 5.55 to − 4.73 kcal/mol. The docking analysis helps strengthen the protein–ligand bond, suggesting a strong binding affinity between ligands and spike protein RBD. The pharmacokinetics of these compounds showed their appropriate drug-like properties with no toxicity and allergenicity. The compounds have good bioavailability cannot penetrate the Blood–brain barrier and cannot elicit an immune response especially **A3, A4, C3, C6A, C6B, C6C, C6E, C6H, C6I,** and **C6J** which can not violate Lipinski’s rule having good pharmacokinetics properties. Based on good docking score, binding position, and pharmacokinetics properties, these compounds were further subjected to in vitro inhibition assay to confirm their drug candidacy. Fortunately, nine new inhibitors were identified against the SARS CoV-2 spike protein. Among them, **C3**, **C**-**6C**, **C6E and C6D,** exhibited higher anti spike protein capability with 91.00%, 90.00%, 87.00%, and 86.20%, inhibition. Acetyl 11-keto-β-boswellic acid has been recognized as a potential natural product for the treatment of various viral illnesses^26,92^. *Boswellia* plays a crucial role in various diseases, for instance, its serrata gum resin is already reported in inflammation, chikungunya, and vesicular stomatitis and β-boswellic acids are also reported against HSV-1, HIV, and herpes virus^93–96^. In the recent COVID-19 pandemic, boswellic acids with glycyrrhizin (GR) combination displayed successful actions against COVID-19^97,98^. Due to high inhibition potential against spike protein RBD the top compounds **C3** and **C6E** docking interaction were confirmed by molecular dynamic simulation. The results of binding free energy calculations demonstrated that compounds **C3** and **C6E** displayed the most elevated energy values, with values of ΔG_TOTAL_ − 38.0 ± 0.08 kcal/mol and − 41.98 ± 0.08 kcal/mol respectively. Based on the medicinal implications of AKBA, and good inhibitory sresults in our current findings, these newly identified spike protein inhibitors appeal further investigation to be used as possible drug candidates to tackle this severe infection (Supplementary Information).

## Conclusions

An international health problem exists because of the SARS-CoV-2-caused COVID-19 pandemic. Scientists’ current efforts are directed toward the development of therapeutics for this devastating pandemic. Because the virus’ spike protein interacts with the host cell's *h*ACE2 receptor, this interaction has been identified as a potential site for therapeutic development. After discovering that acetyl 11-keto-boswellic acid (AKBA) is a potential compound for spike protein through virtual screening and inhibitory tests, we scanned the remaining AKBA derivatives in our in-house database using 2D-similarity searching. Subsequently, 19 compounds were chosen based on their homology to AKBA (> 85%) and docked with the spike protein’s receptor binding domain (RBD). Eleven compounds (**A2-A3, A7, C3, C6B, C6C, C6D, C6E, C6G, C6J,** and **C6K)** showed considerable inhibitory activity with good percent inhibition (range: > 72–90). Our in-silico results were subsequently corroborated by in vitro bioassay. Particularly impressive anti-SARS CoV-2 spike protein activities were shown by **C3** (90.00%), **C6E** (91.00%), **C-6C** (87.00%), and **C6D** (86.20%). The docking interaction of highly inhibitory potential potent compounds have further confirm by MD simulation which give us a good binding energy.

### Supplementary Information

Supplementary Information.

## Data Availability

All data generated or analyzed during this study are included in this published article.
