# Supplementary material for: Identification of new pharmacophore against SARS-CoV-2 spike protein by multi-fold computational and biochemical techniques
Source: Sci Rep. 2024 Feb 13;14:3590. doi: 10.1038/s41598-024-53911-6 (PMC10864406; doi:10.1038/s41598-024-53911-6)
Supplement: Supplementary file 1 — Supplementary Information. [file 41598_2024_53911_MOESM1_ESM.docx]

**Identification of new pharmacophore against SARS-CoV-2 spike protein by multi-fold computational and biochemical techniques.**

Atta Ullah^a^, Saeed Ullah^a^, Sobia Ahsan Halim^a^, Muhammad Waqas^a^, Basharat Ali^b^, Farid S. Ataya^c^, Nasser M. El-Sabbagh^d^, Gaber El-Saber Batiha^e^, Satya Kumar Avula^a^, Rene Csuk^f^, Ajmal Khan^a^*, Ahmed Al-Harrasi^a^*

^a^Natural and Medical Sciences Research Center, University of Nizwa, Birkat-ul-Mouz 616, Nizwa, Sultanate of Oman

^b^Sulaiman Bin Abdullah Aba Al-Khail – Center for Interdisciplinary Research in Basic Sciences (SA-CIRBS), International Islamic University, Islamabad, Pakistan

^c^Department of Biochemistry, College of Science, King Saud University, PO Box 2455, Riyadh 11451, Saudi Arabia.

^d^Department of Veterinary Pharmacology, Faculty of Veterinary Medicine, Alexandria University, Edfina, Egypt.

^e^Department of Pharmacology and Therapeutics, Faculty of Veterinary Medicine, Damanhour University, Damanhour 22511, AlBeheira, Egypt

^f^Organic Chemistry, Martin-Luther-University Halle-Wittenberg, Kurt-Mothes-Str. 2, d-06120, Halle (Saale), Germany

Correspondence:

**Dr. Ajmal Khan**

Natural and Medical Sciences Research Center, University of Nizwa, P.O Box 33, Postal Code 616, Birkat Al Mauz, Nizwa, Sultanate of Oman. Email: ajmalkhan@unizwa.edu.om (A.K);

**Prof. Dr. Ahmed Al-Harrasi**

Natural and Medical Sciences Research Center, University of Nizwa, P.O Box 33, Postal Code 616, Birkat Al Mauz, Nizwa, Sultanate of Oman. Email: aharrasi@unizwa.edu.om (A.A-H)

**Table of content**

| **Table S1** | Docking interaction of AKBA and selected 19 compounds with spike protein. |
| --- | --- |
| **Table S2** | Pharmacokinetics properties of AKBA and its selected derivatives. |
| **Table S3** | Toxicity profile of selected compounds computed using the Pro Tox-II server. IAV inactive, AV Active |
| **Table S4** | Drug-likeness and medicinal property assessment of selected inhibitors compounds against spike protein RBD via SwissADME server. |
| **Table S5** | Hydrogen bond analysis of selected inhibitor compounds C3 and C6E |
| **Figure S1** | Root mean square fluctaution analysis of selected protei 6MOJ and inhibitor compounds C3 and C6E. |

**Table S1.** Docking interaction of AKBA and selected 19 compounds with spike protein.

| **Compounds** | **Docking score (Kcal/mol)** | **Ligand atoms** | **Receptor atoms** | **Residues** | **Interaction Type** | **Distance (Å)** | **Energy (kcal/mol)** |
| --- | --- | --- | --- | --- | --- | --- | --- |
| **AKBA** | -5.8732 | O75 | OH | TYR505 | HBA | 2.06 | -0.7 |
|  |  | 081 | NE2 | GLN409 | HBA | 3.09 | -1.2 |
|  |  | O31 | OH | TYR473 | HBA | 2.14 | -1.5 |
| **A1** | -5.1497 | O72 | OH | TYR453 | HBA | 3.19 | -0.6 |
|  |  | O86 | NH | ARG403 | HBA | 2.98 | -0.8 |
| **A2** | -4.7336 | O83 | O | SER494 | HBD | 3.08 | -1.2 |
|  |  | O86 | OH | TYR453 | HBA | 2.96 | -1.7 |
| **A3** | -4.7440 | N41 | N | GLY496 | HBA | 3.14 | -0.7 |
|  |  | N43 | ND2 | ASN501 | HBA | 3.36 | -0.8 |
| **A4** | -5.0870 | O49 | N | GLY496 | HBA | 2.92 | -2.1 |
|  |  | 6-ring | NG1 | THR500 | π-H | 4.25 | -0.5 |
| **A5** | -5.2517 | N39 | ND2 | ASN501 | HBA | 3.23 | -0.6 |
|  |  | N39 | N | GLY496 | π -H | 3.74 | -3.2 |
|  |  | N39 | CD2 | TYR505 | π -H | 4.33 | -0.5 |
| **A6** | -5.4260 | C34 | O | GLY496 | HBD | 3.14 | -1.1 |
|  |  | O32 | N | GLY496 | HBA | 2.75 | -2.8 |
|  |  | O50 | OH | TYR449 | HBA | 3.36 | -1.0 |
| **A7** | -4.7721 | N85 | N | GLY504 | HBA | 3.04 | -3.2 |
|  |  | O90 | N | VAL503 | HBA | 2.85 | -4.3 |
| **C3** | -5.4451 | O70 | N | GLY496 | HBA | 2.73 | -4.4 |
|  |  | O80 | OH | TYR505 | HBA | 2.71 | -4.0 |
| **C6A**  **C6B** | -4.9335 | O32 | N | GLY496 | HBA | 3.09 | -2.6 |
|  |  | O48 | OH | TYR505 | HBA | 3.00 | -2.5 |
| **C6C**  **C6D**  **C6E**  **C6F** | -5.3182 | O32 | OH | TYR453 | HBA | 2.82 | -2.7 |
|  |  | N38 | OH | TYR505 | HBA | 2.92 | -0.5 |
|  |  | N39 | OH | TYR505 | HBA | 3.00 | -0.7 |
|  |  | O40 | OH | TYR449 | HBA | 2.80 | -2.5 |
| **C6G**  **C6H** | -5.7291 | O70 | OH | TYR449 | HBA | 2.77 | -3.5 |
|  |  | O97 | OH | TYR453 | HBA | 2.73 | -2.9 |
| **C6I**  **C6J** | -5.3292 | O75 | NH1 | ARG403 | HBA | 2.94 | -3.1 |
|  |  | F106 | N | GLY496 | HBA | 2.96 | -0.5 |
|  |  | 5-ring | OH | TYR505 | π -H | 3.92 | -0.8 |
| **C6A**  **C6B** | -5.4171 | O32 | OH | TYR505 | HBA | 2.74 | -2.8 |
|  |  | O40 | OH | TYR453 | HBA | 2.93 | -1.5 |
| **C6C**  **C6D**  **C6E** | -5.5495 | O32 | OH | TYR449 | HBA | 2.79 | -2.4 |
|  |  | N39 | N | GLY496 | HBA | 3.30 | -0.9 |
|  |  | 5-ring | CA | TYR495 | π -H | 4.21 | -0.5 |
| **C6F**  **C6G** | -5.6146 | O32 | OH | TYR 449 | HBA | 2.70 | -3.1 |
|  |  | 5-ring | CA | TYR495 | π -H | 4.17 | -0.9 |
| **C6H**  **C6I** | -5.2718 | O32 | N | GLY502 | HBA | 2.84 | -5.6 |
|  |  | O40 | OG1 | THR500 | HBA | 2.69 | -1.3 |
| **C6J**  **C6A** | -5.2293 | O32 | OH | TYR449 | HBA | 2.72 | -3.7 |
|  |  | 5-ring | CA | TYR495 | π -H | 4.18 | -0.7 |
|  |  | 5-ring | CE2 | TYR505 | π -H | 4.12 | -0.7 |
| **C6B**  **C6C** | -5.1829 | O32 | OG1 | THR500 | HBA | 2.88 | -2.5 |
|  |  | O40 | OH | TYR449 | HBA | 2.76 | -2.8 |
| **C6D** | -4.6723 | N39 | N | GLY496 | HBA | 3.13 | -1.2 |
|  |  | 5-ring | CA | TYR495 | π -H | 4.14 | -0.6 |
|  |  | 5-ring | CE2 | TYR505 | π -H | 4.39 | -0.5 |
| ***HBA*** *= Hydrogen bond acceptor,* ***HBD*** *= Hydrogen bond donor,* ***π -H****= Pi-Hydrogen bond* | | | | | | | |

**Table S2**. Pharmacokinetics properties of AKBA and its selected derivatives.

| **Names** |  | **Pharmacokinetics** | | | | | | |
| --- | --- | --- | --- | --- | --- | --- | --- | --- |
|  | **GIA** | **Bioavailability score** | **BBBP** | **P-gp sub** | **CYP1A2 inhibitor** | **CYP2C19 inhibitor** | **CYP2C9 inhibitor** | **CYP2D6 inhibitor** |
| **AKBA** | High | 0.17 | NO | NO | NO | NO | NO | NO |
| **A1** | Low | 0.17 | NO | NO | NO | NO | NO | NO |
| **A2** | High | 0.17 | NO | NO | NO | NO | NO | NO |
| **A3** | High | 0.17 | NO | YES | NO | NO | NO | NO |
| **A4** | Low | 0.17 | NO | NO | NO | NO | NO | Yes |
| **A5** | Low | 0.17 | NO | NO | NO | NO | NO | NO |
| **A6** | Low | 0.17 | NO | NO | NO | NO | NO | Yes |
| **A7** | Moderate | 0.17 | NO | Yes | NO | NO | NO | Yes |
| **C3** | High | 0.19 | NO | NO | NO | NO | NO | NO |
| **C6A** | Low | 0.17 | NO | NO | NO | NO | NO | Yes |
| **C6B** | Moderate | 0.17 | NO | NO | NO | NO | NO | NO |
| **C6C** | High | 0.17 | NO | NO | NO | NO | NO | NO |
| **C6D** | High | 0.17 | NO | NO | NO | NO | NO | Yes |
| **C6E** | High | 0.19 | NO | NO | NO | NO | NO | NO |
| **C6F** | Low | 0.17 | NO | NO | NO | NO | NO | NO |
| **C6G** | Low | 0.17 | NO | NO | NO | NO | NO | Yes |
| **C6H** | Low | 0.17 | NO | NO | NO | NO | NO | NO |
| **C6I** | High | 0.17 | NO | NO | NO | NO | NO | NO |
| **C6J** | High | 0.19 | NO | NO | NO | NO | NO | NO |
| **C6K** | High | 0.17 | NO | NO | NO | NO | NO | Yes |
|  | **GIA** = gastrointestinal absorption, **BBBP** = Blood brain barrier permeability, **P-gp** **Sub** = P-glycoprotein substrate | | | | | | | |

**Table S3.** Toxicity profile of selected compounds computed using the Pro Tox-II server. IAV inactive, AV Active

| Codes | Predicted toxicity class | LD50(mg/Kg) | Organ toxicity | Toxicity end points | | | |
| --- | --- | --- | --- | --- | --- | --- | --- |
|  |  |  | Hepato-toxicity | Carcinogenicity | Immuno-toxicity | Mutagenicity | Cytotoxicity |
| **AKBA** | 4 | 1190 | IAV | IA | IAV | IAV | IAV |
| **A1** | 4 | 1130 | IAV | IAV | AV | AV | IAV |
| **A2** | 5 | 3000 | IAV | IAV | IAV | IAV | IAV |
| **A3** | 4 | 1130 | IAV | IAV | IAV | IAV | IAV |
| **A4** | 4 | 1130 | IAV | IAV | AV | IAV | IAV |
| **A5** | 4 | 1500 | IAV | IAV | AV | IAV | AV |
| **A6** | 5 | 1700 | IAV | IAV | AV | IAV | IAV |
| **A7** | 4 | 1230 | IAV | IAV | IAV | IAV | IAV |
| **C3** | 5 | 3300 | IAV | IAV | IAV | IAV | IAV |
| **C6A** | 4 | 500 | IAV | IAV | IAV | IAV | IAV |
| **C6B** | 4 | 550 | IAV | IAV | IAV | IAV | IAV |
| **C6C** | 4 | 500 | IAV | IAV | IAV | IAV | IAV |
| **C6D** | 4 | 500 | IAV | IAV | IAV | IAV | IAV |
| **C6E** | 4 | 500 | IAV | IAV | IAV | IAV | IAV |
| **C6F** | 4 | 500 | IAV | IAV | IAV | IAV | IAV |
| **C6G** | 4 | 500 | IAV | IAV | AV | IAV | IAV |
| **C6H** | 4 | 500 | IAV | IAV | AV | IAV | IAV |
| **C6I** | 4 | 500 | IAV | IAV | AV | IAV | IAV |
| **C6J** | 4 | 500 | IAV | IAV | IAV | IAV | IAV |
| **C6K** | 4 | 500 | IAV | IAV | IAV | IAV | AV |

**Table S4:** Drug-likeness and medicinal property assessment of selected inhibitors compounds against spike protein RBD via SwissADME server.

| Codes | Drug likeness | | | | | Medicinal Chemistry | | | |
| --- | --- | --- | --- | --- | --- | --- | --- | --- | --- |
|  | Lipinski | Ghose | Veber | Egan | Muegge | PAINS | Structural  Alert | Lead likeness | Synthetic accessibility  score |
| AKBA | 1 violation: MW>500 | Yes | Yes | Yes | Yes | 0 alert | 0 alert | Yes | 3.24 |
| A1 | 1 violation: MW>500 | Yes | Yes | Yes | Yes | 0 alert | 0 alert | Yes | 3.37 |
| A2 | 1 violation: MW>500 | No; 1 violation: MR>130 | Yes | 1 violation: WLOGP>500 | Yes | 0 alert | 0 alert | Yes | 3.19 |
| A3 | 1 violation: MW>500 | Yes | Yes | Yes | Yes | 0 alert | 0 alert | No; 1 violation: XLOGP>3.5 | 3.99 |
| A4 | 1 violation: MW>500 | Yes | Yes | Yes | Yes | 0 alert | 0 alert | No; 1 violation: MW>350 | 4.43 |
| A5 | 1 violation: MW>500 | Yes | Yes | 1 violation: WLOGP>500 | Yes | 0 alert | 0 alert | No; 1 violation: MW>350 | 3.99 |
| A6 | 1 violation: MW>500 | No; 1 violation: MR>130 | Yes | Yes | Yes | 0 alert | 0 alert | No; 1 violation: MW>350 | 4.43 |
| A7 | 1 violation: MW>500 | Yes | Yes | Yes | Yes | 0 alert | 0 alert | No; 1 violation: MW>350 | 4.36 |
| C3 | 1 violation: MW>500 | Yes | Yes | Yes | Yes | 0 alert | 0 alert | Yes | 3.14 |
| C6A | 1 violation: MW>500 | Yes | Yes | Yes | Yes | 0 alert | 0 alert | No; 1 violation: MW>350 | 3.32 |
| C6B | Yes; 0 violation | Yes | Yes | Yes | Yes | 0 alert | 0 alert | No; 1 violation: MW>350 | 4.41 |
| C6C | 1 violation: MW>500 | Yes | Yes | Yes | Yes | 0 alert | 0 alert | No; 2 violations: MW>350, Rotors>7 | 3.93 |
| C6D | 1 violation: MW>500 | Yes | Yes | Yes | Yes | 0 alert | 0 alert | No; 1 violation: MW>350 | 4.56 |
| C6E | 1 violation: MW>500 | Yes | Yes | Yes | Yes | 0 alert | 0 alert | No; 1 violation: Rotors>7 | 3.63 |
| C6F | 1 violation: MW>500 | Yes | Yes | 1 violation: WLOGP>500 | Yes | 0 alert | 0 alert | Yes | 4.56 |
| C6G | 1 violation: MW>500 | Yes | Yes | 1 violation: WLOGP>500 | Yes | 0 alert | 0 alert | No; 2 violations: MW>350, Rotors>7 | 3.90 |
| C6H | 1 violation: MW>500 | No; 1 violation: MR>130 | Yes | Yes | Yes | 0 alert | 0 alert | No; 2 violations: MW>350, Rotors>7 | 4.08 |
| C6I | 1 violation: MW>500 | Yes | Yes | Yes | Yes | 0 alert | 0 alert | No; 2 violations: MW>350, Rotors>7 | 4.21 |
| C6J | 1 violation: MW>500 | Yes | Yes | Yes | Yes | 0 alert | 0 alert | No; 1 violation: Rotors>7 | 4.27 |
| C6K | 1 violation: MW>500 | Yes | Yes | Yes | Yes | 0 alert | 0 alert | No; 1 violation: MW>350 | 3.31 |

**Table S5**. Hydrogen bond analysis of selected inhibitor compounds C3 and C-6E

| **Complex** | **Acceptor Atoms** | **Donor Atoms** | **Bond Life** | **Distance (****Å)** | **Angle (Å ˚)** |
| --- | --- | --- | --- | --- | --- |
| 6M0J | 166-OE1 | ASN115 | 81.20% | 2.83 | 0.09 |
|  | [155-OD1](mailto:155@OD1) | GLN166 | 79.00% | 2.75 | 0.12 |
|  | 170-N | GLY170 | 78.40% | 2.86 | 0.08 |
|  | 161-OE1 | GLY164 | 77.90% | 2.81 | 0.10 |
|  | [164-O](mailto:164@O) | GLN161 | 72.00% | 2.83 | 0.10 |
|  | [117-OH](mailto:117@OH) | TYR117 | 61.50% | 2.70 | 0.12 |
|  | [173-OH](mailto:173@OH) | THR168 | 58.70% | 2.73 | 0.12 |
|  | 168-OG1 | GLY171 | 37.50% | 2.87 | 0.10 |
|  | 155-ND2 | ASN155 | 32.60% | 2.86 | 0.09 |
|  | 117-OH | TYR117 | 31.70% | 2.71 | 0.12 |
|  | 114-O | GLY114 | 30.20% | 2.84 | 0.09 |
|  | 85-NZ | LYS185 | 28.60% | 2.79 | 0.10 |
|  | 121-OH | GLN161 | 11.00% | 2.87 | 0.09 |
|  | 168-O | ASN155 | 0.65% | 2.88 | 0.09 |
| C3 | C3-195-O2 | GLY-170-N | 71.95% | 2.87 | 160.08 |
|  | C3-195-O | GLY-164-N | 42.30% | 2.86 | 146.99 |
|  | C3-195-O4 | GLN-161-NE2 | 16.80% | 2.88 | 157.24 |
|  | C3-195-O2 | ASN-169-ND2 | 15.90% | 2.85 | 151.97 |
|  | C3-195-O | GLN-161-NE2 | 4.60% | 2.87 | 151.13 |
|  | C3-195-O2 | GLN-161-NE2 | 4.50% | 2.88 | 156.46 |
|  | C3-195-O4 | TYR-117-OH | 4.00% | 2.76 | 162.06 |
|  | C3-195-O | TYR-117-OH | 2.80% | 2.77 | 159.65 |
|  | C3-195-O2 | GLN-166-NE2 | 2.40% | 2.87 | 155.54 |
|  | C3-195-O | GLN-161-NE2 | 2.00% | 2.86 | 154.52 |
|  | C3-195-O4 | GLN-161-NE2 | 0.18% | 2.86 | 155.73 |
|  | C3-195-O4 | GLN-166-NE2 | 0.17% | 2.84 | 154.86 |
|  | C3-195-O2 | GLN-166-NE2 | 0.17% | 2.88 | 153.47 |
|  | C3-195-O2 | GLN-161-NE2 | 0.10% | 2.87 | 154.95 |
|  | C3-195-O4 | ARG-71-NH1 | 0.08% | 2.86 | 153.70 |
|  | C3-195-O | ARG-71-NH1 | 0.08% | 2.86 | 148.19 |
|  | C3-195-O4 | GLN-166-NE2 | 0.08% | 2.94 | 160.87 |
|  | C3-195-O | TYR-173-OH | 0.05% | 2.80 | 154.13 |
|  | C3-195-O | THR-168-OG1 | 0.05% | 2.81 | 150.38 |
|  | C3-195-O4 | TYR-173-OH | 0.03% | 2.88 | 149.17 |
|  | C3-195-O | GLN-166-NE2 | 0.02% | 2.82 | 143.35 |
|  | C3-195-O2 | TYR-173-OH | 0.02% | 2.83 | 153.21 |
|  | C3-195-O4 | ARG-71-NH2 | 0.02% | 2.84 | 147.19 |
|  | C3-195-O | TYR-121-OH | 0.02% | 2.85 | 160.62 |
|  | [C3-195-O2](mailto:*_195@O2) | ARG-71-NH1 | 0.01% | 2.82 | 151.66 |
| C6E | C6-195-N2 | THR-138-OG1 | 65.24% | 2.87 | 157.89 |
|  | C6-195-N2 | GLN-166-NE2 | 39.00% | 2.93 | 160.35 |
|  | C6-195-N1 | SER-162-OG | 33.00% | 2.85 | 156.24 |
|  | C6-195-N2 | GLN-166-NE2 | 31.00% | 2.93 | 159.32 |
|  | C6-195-N1 | THR-138-OG1 | 24.00% | 2.90 | 148.03 |
|  | C6-195-O5 | THR-138-N | 20.30% | 2.90 | 156.80 |
|  | C6-195-N2 | TYR-19-OH | 17.00% | 2.89 | 159.72 |
|  | C6-195-N2 | THR-168-OG1 | 1.50% | 2.91 | 156.47 |
|  | C6-195-O1 | TYR-19-OH | 1.20% | 2.86 | 161.30 |
|  | C6-195-N1 | TYR-19-OH | 0.10% | 2.88 | 153.41 |
|  | C6-195-N1 | GLN-166-NE2 | 0.10% | 2.91 | 155.81 |
|  | C6-195-O4 | THR-138-OG1 | 0.06% | 2.81 | 163.18 |
|  | C6-195-N2 | ASN-118-ND2 | 0.06% | 2.91 | 146.80 |
|  | C6-195-N2 | ARG-14-NH1 | 0.05% | 2.93 | 149.27 |
|  | C6-195-O5 | GLN-161-NE2 | 0.05% | 2.88 | 159.31 |
|  | C6-195-O4 | GLY-170-N | 0.05% | 2.92 | 144.15 |
|  | C6-195-O5 | GLU-152-N | 0.04% | 2.88 | 160.24 |
|  | C6-195-O5 | ASN-118-ND2 | 0.03% | 2.85 | 161.20 |
|  | C6-195-N2 | SER-162-OG | 0.03% | 2.87 | 155.57 |
|  | C6-195-O2 | ASN-118-ND2 | 0.03% | 2.91 | 155.49 |
|  | C6-195-N2 | ARG-14-NH2 | 0.03% | 2.92 | 166.49 |
|  | C6-195-O5 | TYR-117-N | 0.03% | 2.93 | 150.22 |
|  | C6-195-O4 | ARG-14-NH1 | 0.03% | 2.94 | 161.31 |
|  | C6-195-N1 | GLN-166-NE2 | 0.03% | 2.95 | 155.74 |
|  | C6-195-N2 | SER-162-N | 0.03% | 2.96 | 143.80 |
|  | C6-195-O5 | LYS-112-NZ | 0.02% | 2.85 | 148.78 |
|  | C6-195-O5 | THR-138-OG1 | 0.02% | 2.86 | 140.64 |
|  | C6-195-O2 | TYR-19-OH | 0.02% | 2.90 | 143.57 |
|  | C6-195-N1 | TYR-117-OH | 0.02% | 2.92 | 157.70 |
|  | C6-195-N1 | SER-162-N | 0.02% | 2.93 | 156.30 |
|  | C6-195-N1 | GLY-114-N | 0.02% | 2.97 | 157.61 |
|  | C6-195-O5 | SER-162-OG | 0.02% | 2.98 | 146.07 |
|  | C6-195-O4 | TYR-19-OH | 0.01% | 2.66 | 158.62 |
|  | C6-195-O5 | TYR-117-OH | 0.01% | 2.74 | 151.08 |
|  | C6-195-O5 | LYS-112-NZ | 0.01% | 2.76 | 136.52 |
|  | C6-195-O2 | ASN-118-ND2 | 0.01% | 2.81 | 152.31 |
|  | C6-195-O4 | ARG-14-NH2 | 0.01% | 2.82 | 176.42 |
|  | C6-195-O2 | ARG-14-NH2 | 0.01% | 2.87 | 165.76 |
|  | C6-195-O1 | ASN-118-ND2 | 0.01% | 2.93 | 147.50 |
|  | C6-195-N1 | ARG-14-NH1 | 0.01% | 2.94 | 154.84 |
|  | C6-195-N2 | ARG-14-NH1 | 0.01% | 2.96 | 164.55 |
|  | C6-195-O4 | ASN-22-ND2 | 0.01% | 2.96 | 146.38 |
|  | C6-195-O2 | ARG-14-NH2 | 0.01% | 2.97 | 146.57 |
|  | C6-195-N2 | TYR-117-OH | 0.01% | 2.97 | 161.69 |
|  | C6-195-O5 | SER-162-N | 0.01% | 2.98 | 158.29 |
|  | C6-195-O4 | GLU-152-N | 0.01% | 2.99 | 142.44 |


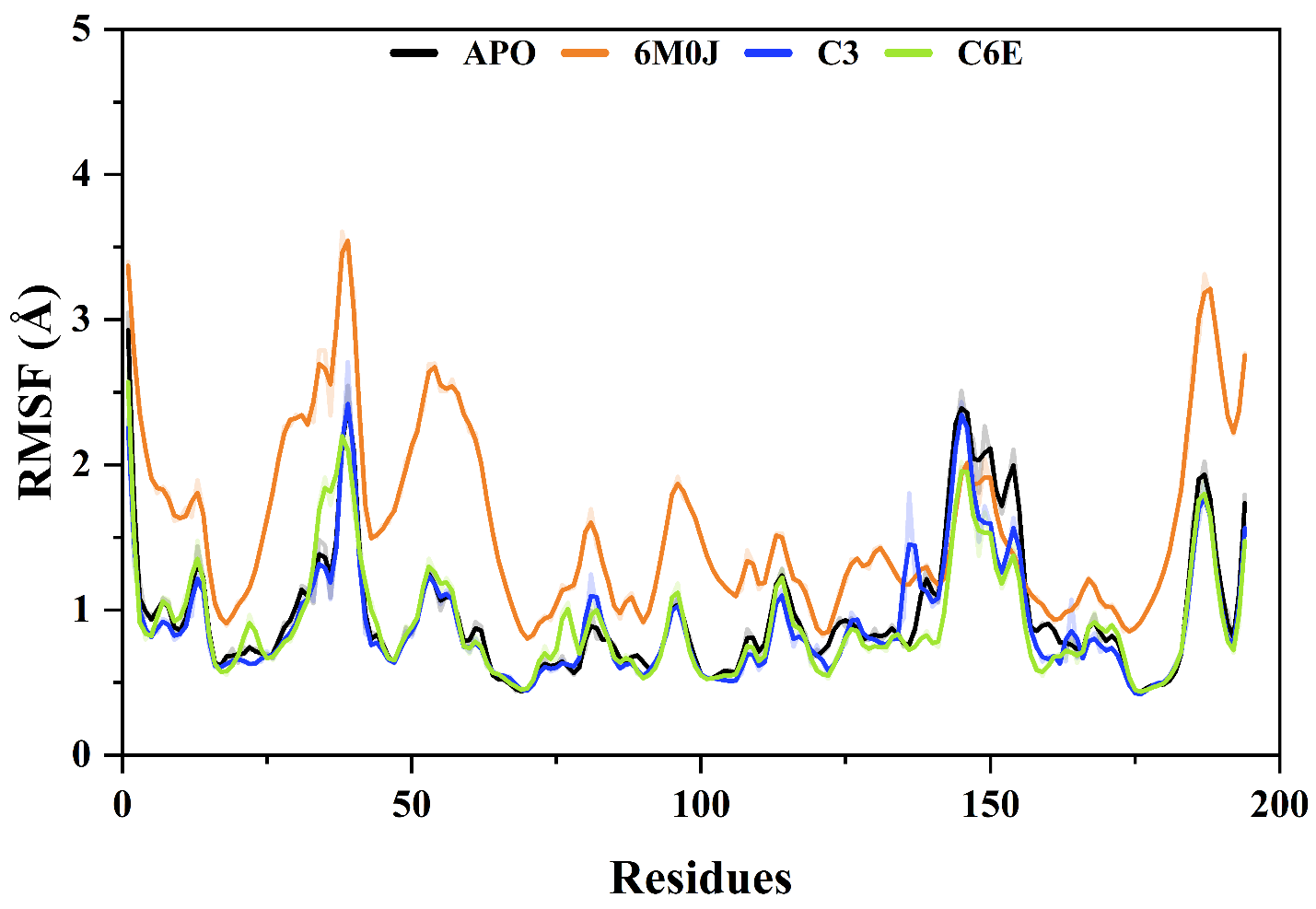


**Figure S1**. Root mean square fluctaution analysis of selected protei 6MOJ and inhibitor compounds C3 and C6E.
